# Environmental microcystin targets the microbiome and increases the risk of intestinal inflammatory pathology via NOX2 in underlying murine model of Nonalcoholic Fatty Liver Disease

**DOI:** 10.1038/s41598-019-45009-1

**Published:** 2019-06-19

**Authors:** Sutapa Sarkar, Diana Kimono, Muayad Albadrani, Ratanesh K. Seth, Philip Busbee, Hasan Alghetaa, Dwayne E. Porter, Geoff I. Scott, Bryan Brooks, Mitzi Nagarkatti, Prakash Nagarkatti, Saurabh Chatterjee

**Affiliations:** 1 Environmental Health and Disease Laboratory, Department of Environmental Health Sciences, University of South Carolina, Columbia, USA; 2 NIEHS Center for Oceans and Human Health on Climate Change Interactions, Department of Environmental Health Sciences, University of South Carolina, Columbia, USA; 3 Department of Environmental Science, Baylor University, Waco, USA; 4 Pathology, Microbiology and Immunology, University of South Carolina School of Medicine, Columbia, USA

**Keywords:** Non-alcoholic fatty liver disease, Dysbiosis

## Abstract

With increased climate change pressures likely to influence harmful algal blooms, exposure to microcystin, a known hepatotoxin and a byproduct of cyanobacterial blooms can be a risk factor for NAFLD associated comorbidities. Using both *in vivo* and *in vitro* experiments we show that microcystin exposure in NAFLD mice cause rapid alteration of gut microbiome, rise in bacterial genus known for mediating gut inflammation and lactate production. Changes in the microbiome were strongly associated with inflammatory pathology in the intestine, gut leaching, tight junction protein alterations and increased oxidative tyrosyl radicals. Increased lactate producing bacteria from the altered microbiome was associated with increased NOX-2, an NADPH oxidase isoform. Activationof NOX2 caused inflammasome activation as shown by NLRP3/ASCII and NLRP3/Casp-1 colocalizations in these cells while use of mice lacking a crucial NOX2 component attenuated inflammatory pathology and redox changes. Mechanistically, NOX2 mediated peroxynitrite species were primary to inflammasome activation and release of inflammatory mediators. Thus, in conclusion, microcystin exposure in NAFLD could significantly alter intestinal pathology especially by the effects on microbiome and resultant redox status thus advancing our understanding of the co-existence of NAFLD-linked inflammatory bowel disease phenotypes in the clinic.

## Introduction

The prevalence of non-alcoholic fatty liver disease (NAFLD) is increasing proportionately^1^. Fatty liver disease is defined as a benign and non-phenotypic (micro vesicular and macro vesicular) accumulation of fat in the liver which often remains asymptomatic for years and assumes a role of a silent killer since it takes years to develop into a potentially full blown disease^2,3^. Following a second hit or as is proclaimed in recent years, multiple hits can progress from a condition of fatty liver (NAFLD) to a more progressive inflammatory condition called non-alcoholic steatohepatitis (NASH)^4,5^. NASH is a potentially progressive liver disease that can lead to cirrhosis, hepatocellular carcinoma, liver transplantation, and death. NAFLD and subsequent NASH is also associated with extrahepatic manifestations such as chronic kidney disease, cardiovascular disease and sleep apnoea^6^. Interestingly patients with type 2 diabetes or inflammatory bowel disease (IBD) can also have parallel NAFLD-like conditions^7^. NAFLD and its more progressive inflammatory form NASH have similar comorbidities in the intestine or can result in colon polyps^8^.

NASH is often diagnosed by abnormal liver chemistry, imaging studies and liver biopsy. As there are risks of potential complications during liver biopsy, many patients do not opt for liver biopsy^9^. The above fact makes it more likely to become a silent killer since there are no early biomarkers for this disease. The mechanisms that account for disease progression in NASH are still poorly understood. NAFLD and NASH carry a large public health burden and create poor health-related quality of life. An important recent study analyzed the 2010 Nationwide Inpatient Sample to compare outcomes and associations between patients with nonalcoholic fatty liver disease (NAFLD)^10^. Compared with other liver diseases, NAFLD and its inflammatory condition NASH are associated with diverticular, inflammatory bowel, gallstone, and benign pancreatitis disorders when these latter disorders are considered as either the principal or associated diagnoses on discharge. These associations suggest shared mechanisms of pathology between NAFLD/NASH and these benign gastrointestinal disorders^10^. Recent data also highlighted the co-existence of NAFLD/NASH and inflammatory bowel disease; both of which are increasingly prevalent disorders with significant complications and impact on future health burden^11^. In both scenarios the missing links to both NAFLD severity inferred by NASH and intestinal inflammatory co-morbidities remain unclear. The diagnosis and treatment of the pathology associated with NAFLD/NASH and its comorbidities are complicated by the contribution of environmental and genetic factors to the risk of developing a progressive course of disease^6^. Thus, it is important to note that a diseased intestine (or an inflamed one) either in an underlying NAFLD or other chronic liver disease phenotypes can complicate diagnosis and worsen outcome.

In the last decade, a growing body of evidence including reports from our laboratory has emerged, shedding light on the potential impact of environmental pollutants on liver health and, in particular, on NAFLD occurrence and its translation into more progressive form of NASH^12,13^. These contaminants have a great steatogenic and inflammatory potential and need to be considered as tangible NAFLD risk factors^14^. Environmental agents, including endocrine-disrupting chemicals (EDCs), which have been linked to other diseases like obesity, may play a role in NAFLD development and progression in to NASH^15^. Drinking water disinfectants and polychlorobiphenyls have been also linked to progressive forms of NAFLD more often referred to as toxicity associated steatohepatitis or TAH^12^. Recent studies on environmental pollution detail a more discrete threat to public health arising from the large scale harmful algal blooms and the components associated with it. HABs contain cyanobacterial toxins and their secondary metabolites which have been found to have huge toxic potential to human health. Microcystins are secondary metabolites produced by cyanobacteria that act as hepatotoxins in higher organisms^16^. Harmful, bloom-forming cyanobacteria (CyanoHABs) are occurring with increasing regularity in freshwater and marine ecosystems. The most commonly occurring cyanobacterial toxins are the hepatotoxic microcystin and nodularin^17^. Studies also show that theiroccurrence is increasing worldwide owing to climate change and anthropogenic activities^18^. Further, integrated analysis of proteomic, metabolic, histological and cytokine profiles revealed that microcystin significantly inhibited fatty acid β-oxidation and hepatic lipoprotein secretion and promoted hepatic inflammation, resulting in NASH^19^.

Based on the startling evidence of the menace of NAFLD that has assumed pandemic proportions, its progression to NASH and comorbidities and the increasing threat of environmental pollution from cyanobacterial toxins, we test the hypothesis that chronic low dose exposure of microcystin acts as a second hit in the progression of ectopic intestinal manifestations of NAFLD/NASH in susceptible populations. In addition, microcystin exposure-induced NAFLD severity and ectopic inflammation in the intestine arises primarily from its effect on gut microbiome and subsequent inflammatory pathways in the intestine. Using a rodent model of NAFLD, the study describes the mechanisms that lead to progressive inflammatory condition in the intestine, a mostly asymptomatic and under reported consequence in this disease. The results described in this study help advance our understanding the underlying pathology in NASH-associated comorbidities.

## Materials and Methods

### Materials

Microcystin (MC) was purchased from Cayman Chemical Company (Ann Arbor, Michigan). Anti- claudin-2, anti-occludin, anti-HMGB1, anti-F4/80, anti-NLRP3, anti-caspase1, anti-GP91phox and anti-IL1β primary antibodies were purchased from Abcam (Cambridge, MA). Anti-ASC2, anti-p47phox anti-3Nitrotyrosine primary antibody was purchased from Santacruz Biotechnology (Dallas, TX). Species-specific biotinylated conjugated secondary antibodies and Streptavidin-HRP (Vectastain Elite ABC kit) were purchased from Vector Laboratories (Burlingame, CA). Fluorescence-conjugated (Alexa Fluor) secondary antibodies, ProLong Gold antifade mounting media with DAPI were purchased from Thermofisher Scientific (Grand Island, NY) and all other chemicals which were used in this project purchased from sigma only if otherwise specified. Paraffin-embedding of tissue sections on slides were done by AML laboratories (Baltimore, MD). All other chemicals were of analytical grade and were purchased from Sigma Chemical Company.

### Mouse model

#### Experimental models used

Pathogen-free, adult (8 weeks old), male C57BL/6 J (WT) mice and p47 phox gene deleted (*p47phox KO*- B6 (Cg)-Ncflm1J/J) mice (Jackson Laboratories, Ban Harbor, ME) were used in the study. The groups used for the experiment were the wild type mice fed with chow diet only(Lean Control), wild type mice fed with methionine choline deficient- high fat diet only (NAFLD), wild type mice fed with chow diet and then exposed to microcystin(Chow + MC), wild type mice fed with methionine choline deficient diet and then exposed to microcystin(NAFLD + MC), one group of p47 phox KO mice fed with chow diet and exposed with microcystin(*CHOW* + *MC p47phox KO*)and another group of p47 phox KO mice fed with methionine choline deficient diet and then exposed to microcystin(NAFLD + MC *p47phox KO)*. The total number of animals in each group were assessed based on the calculations that ensured enough statistical power of 0.5. There were 8 mice per group that were allocated to their respective cages following the procedure of randomization. A total of 48 mice were used in the experiments for the paper. All mice experiments were carried out based on the approval of the institutional animal ethics committee (IACUC) at the University of South Carolina (Assurance number: D16-00028).

### Diet-induced NAFLD model

**Figure 1:**
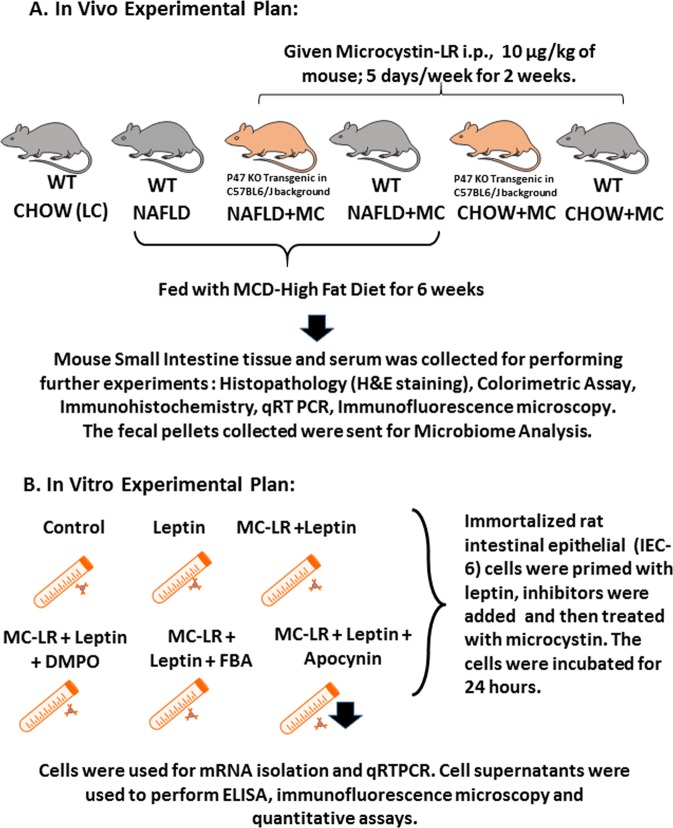
A schematic representation of *in vivo* experimental model and treatments. (**A**) Illustrations of mouse models (grey) with the transgenic mouse (p47 phox illustrated in beige color). (**B**) Cell culture experimental groups illustrated by a tube and cells within. The figure was constructed using the illustration software purchased from Motfolio, USA and applied as per the source attribution guidelines through non exclusive limited license to use illustrations and other products sold through the company.

Pathogen-free, male, C57BL/6 J (WT) and the p47phox gene deleted mice (*p47 phoxKO*) were fed with methionine choline deficient (MCD-HFD) diet from 8 weeks to 14weeks for diet-induced NAFLD model until the experimental dosing was over and were euthanized soon after. The mice weighed about 25 grams during the time of euthanizing. All mice had ad libitum access to food and water and were housed in a temperature controlled room at 23–24 °C with a 12-hour light/dark cycle. All animals were treated in strict accordance with the NIH Guide for the Humane Care and Use of Laboratory Animals and local IACUC standards. We and others have reported previously about the role of NADPH oxidase in the pathology of NASH. NADPH oxidase is comprised of several cytoplasmic and membrane subunits which align on the membrane for an activated enzyme system. P47 phox is the cytosolic subunit of NADPH oxidase and a gene deleted P47 phox mouse was used to ascertain the role of the enzyme in the pathology of microcystin-induced progression of intestinal comorbidities inNASH. A detailed experimental design is described in Fig. 1A,B.

### Exposure of NAFLD mice to environmental toxin microcystin

Wild-type control and gene specific knockout mice (*p47phox KO*), fed with MCD-HFD diet for 6 weeks were then administered with microcystin (10 µg per kg of mouse, 5 dosages per week), through the intraperitoneal route for two weeks. The dosage was given each afternoon at the same time to eliminate any bias in the study. Since this is a sub-chronic study, the cumulative effect of the microcystin on inflammatory phenotypes was considered. The mice were euthanized at the completion of dosage and serum and small intestine tissue was collected for further processing.

### Microbiome analysis

#### 16S rRNA gut microbiota profiling

16S rRNA gut microbial profiling was done on fecal pellets and luminal contents which were collected immediately after euthanasia and stored at −80 °C. For 16S rRNA sequencing, genomic DNA was extracted from 100 mg of the fecal pellets and luminal contents by using the QIAamp DNA Stool Mini Kit (Qiagen, Valencia, CA) according to instructions from the manufacturer. DNA libraries were prepared by amplification of the 16S rRNA V3-V4 hypervariable region with added Illumina adapter overhang nucleotide sequences and sequencing with Illumina (San Diego, CA) MiSeq platform. Sequenced reads were than analyzed using Nephele (https://nephele.niaid.nih.gov), an open-source analysis tool provided by the National Institute of Allergy and Infectious Diseases (NIAID) Office of Cyber Infrastructure and Computational Biology (OCICB) in Bethesda, MD^20^. For microbial profiling, QIIME FASTQ paired end with chimera removal, open reference, and SILVA rRNA database project (Silva_99) options were used.

### Intestinal epithelial cell culture and treatments

Immortalized rat intestinal epithelial cell line (IEC-6) was grown and maintained in complete DMEM media containing high glucose, Insulin-Transferrin-Selenium (ITS) and 10% FBS at 37 °C in a humidified atmosphere of 5% CO_2_. After overnight serum starvation (2.0% FBS) the cells were then treated with vehicle (control, C), Rat Leptin (100 µg/mL), Microcystin (100 μg/mL), Apocynin(100 µg/ml), 5,5-Dimethyl-1-pyrroline N-oxide [DMPO; (100 µg/ml)]and phenylboronic acid[FBA;(100 µg/ml)], separately or in combinations for 24 h. The groups for the *in-vitro* cell treatments were cells only (Control), cells + rat Leptin (Leptin), cells + rat leptin + microcystin (Leptin + MC), cells + apocynin + rat leptin + microcystin (Leptin + MC + Apocynin), cells + phenylboronic acid + leptin + microcystin (Leptin + MC + FBA). Upon completion of treatment, cells and supernatant were processed for PCR, ELISA and immunofluorescence imaging. All the *in-vitro* experiments were performed three times (Fig. 1B).

### Laboratory analysis

#### Quantitative real-time polymerase chain reaction (qRTPCR)

mRNA expression in intestinal epithelial cell line was examined by quantitative real-time PCR analysis. Total RNA was isolated from the cultured cells and was purified with the use of RNeasy mini kit columns (Qiagen, Valencia, CA). cDNA was synthesized from purified RNA (1 μg) using iScript cDNA synthesis kit (Bio-rad, Hercules, CA) following the manufacturer’s standard protocol. Real-time qPCR (qRTPCR) was performed with the gene-specific primers using SsoAdvanced SYBR GreenSupermix and CFX96 thermal cycler (Bio-rad, Hercules, CA). Threshold Cycle (Ct) values for the selected genes were normalized against respective samples internal control (18S). Each reaction was carried out in triplicates for each gene and for each sample. The relative fold change was calculated by the 2-ΔΔCt method using cells only as a control. The sequences for the primers used for Real-time PCR are provided in Table 1.

**Table 1:** Real Time PCR Primer Sequence.

Gene	Primer sequence (5′-3′ orientation)
RN_MCP1	F: CACCTCTCAAGCAGAGCACAG
	R: GGGTTCCATGGTGAAGTCAAC

### Detection of HMGB1 release (ELISA)

Immunoreactivity of HMGB1 was detected in the supernatant of the rat intestinal epithelial cell (IEC-6) groups using Rat High Mobility Group Box 1 Protein ELISA kit (Abclonal Biotechnology co., Ltd) following manufacturer’s protocol. Concentrations of HMGB1 (ng/ml) per well was detected from the standard curve.

### Detection of systemic endotoxemia (LAL assay)

Serum Endotoxin(EU/ml) was detected in the mice groups fed with Chow diet treated with microcystin, NAFLD mice, NAFLD mice treated with microcystin and p47phox knockout mice (fed on both Chow and NAFLD diet) treated with microcystin using Pierce LAL Chromogenic Endotoxin Quantitation Kit (Thermo Scientific) following manufacturer’s protocol.

### Histopathology and immunohistochemistry (IHC)

Formalin-fixed, paraffin-embedded, 5 µm thick intestine sections were used for histopathology by standard hematoxylin and eosin (H&E) staining method and IHC staining. The sections were randomly selected from jejunum and ileal sections of the small intestine. Deparaffinization of the sections followed by heat based epitope retrieval and both endogenous peroxidase blocking (3% H_2_O_2_, 5 min) and serum blocking (5% normal serum, 1 h) were carried out. Further, sections were incubated with primary antibody (1:500) for 2 h at RT and then species-specific anti-IgG secondary antibodies and conjugation with HRP was performed using Vectastain Elite ABC kit following manufacturer’s protocols. Finally, 3, 3-Diaminobenzidine (DAB) were used as a chromogen substrate and counter stained with Mayer’s hematoxylin. Morphometric analysis was done using CellSens Software from Olympus America.

### Immuno-fluorescence microscopy

The deparaffinized intestine tissue section from all the mice groups was subjected to epitope retrieval and permeabilization (0.01% triton X-100) followed by blocking with 5% normal serum. Fixed rat intestinal epithelial cells on the cover glass were subjected to permeabilization (0.01% triton X-100) followed by blocking with 10% normal serum. Further, both tissue sections and cells were incubated with primary antibody (1:250) overnight at 4 °C. Species-specific anti-IgG secondary antibody conjugated with Alexa fluor 633 (red) and Alexa fluor 488 (green) was used to observe the antigen-specific immunoreactivity. Sections were mounted in Prolong gold antifade reagent with DAPI (counter stain). Morphometric analysis was done using CellSens Software from Olympus America.

### Detection of ATP release

Rat intestinal epithelial cells were treated with the nitric oxide (NO) donor (DETA-NONOate) separately and with the combination of DMPO (100 µg/ml) (DETA-NONOate + DMPO) and phenylboronic acid (FBA; 100 µg/ml) (DETA-NONOate + FBA). The supernatants were collected and concentration of ATP (µM) per well was detected using Colorimetric ATP Assay kit (from Abcam) following manufacturer’s protocol.

### Detection of nitric oxide

Concentration of nitrite (µM) per well was detected in the rat intestinal epithelial cell supernatants treated with Leptin and Microcystin using Griess reagent system kit (Promega, USA) following manufacturer’s protocol.

### Statistical analyses

All *in vivo* experiments were repeated three times with at least eight mice per group (N = 8); data from each group of eight mice were pooled). Based on our preliminary data conducted prior to the detailed experiments reported in this manuscript, the Type I error rate (α) corresponded to a desired p value for statistical hypothesis testing. For animal numbers the ‘standard’ α value was set up as 0.05. The power of an animal experiment (1-β) and corresponded to the probability of a detecting a result that is a true. Accordingly, power levels were set to 0.8 and all animal numbers were calculated accordingly. All alpha and power values were calculated using the PS software from Vanderbilt University. The statistical analysis was carried out by unpaired t-test and analysis of variance (ANOVA) for assessing difference between multiple groups. For all analysis P < 0.05 was considered statistically significant. For experiments involving 2 groups where distribution of data was not clearly parametric, Mann-Whitney U tests were performed with GraphPad Prism Software Inc, CA, Version 5.03. For experiments involving 3 or more groups, data were evaluated using one-way ANOVA with multiple comparison post hoc analysis. Data are expressed as mean ± SEM, or as absolute number or percentage for categorical variables. The significance level was set at α = 5% for all comparisons.

## Results

### Pathophysiology of non-alcoholic fatty liver disease in mice liver following microcystin exposure

**Figure 2:**
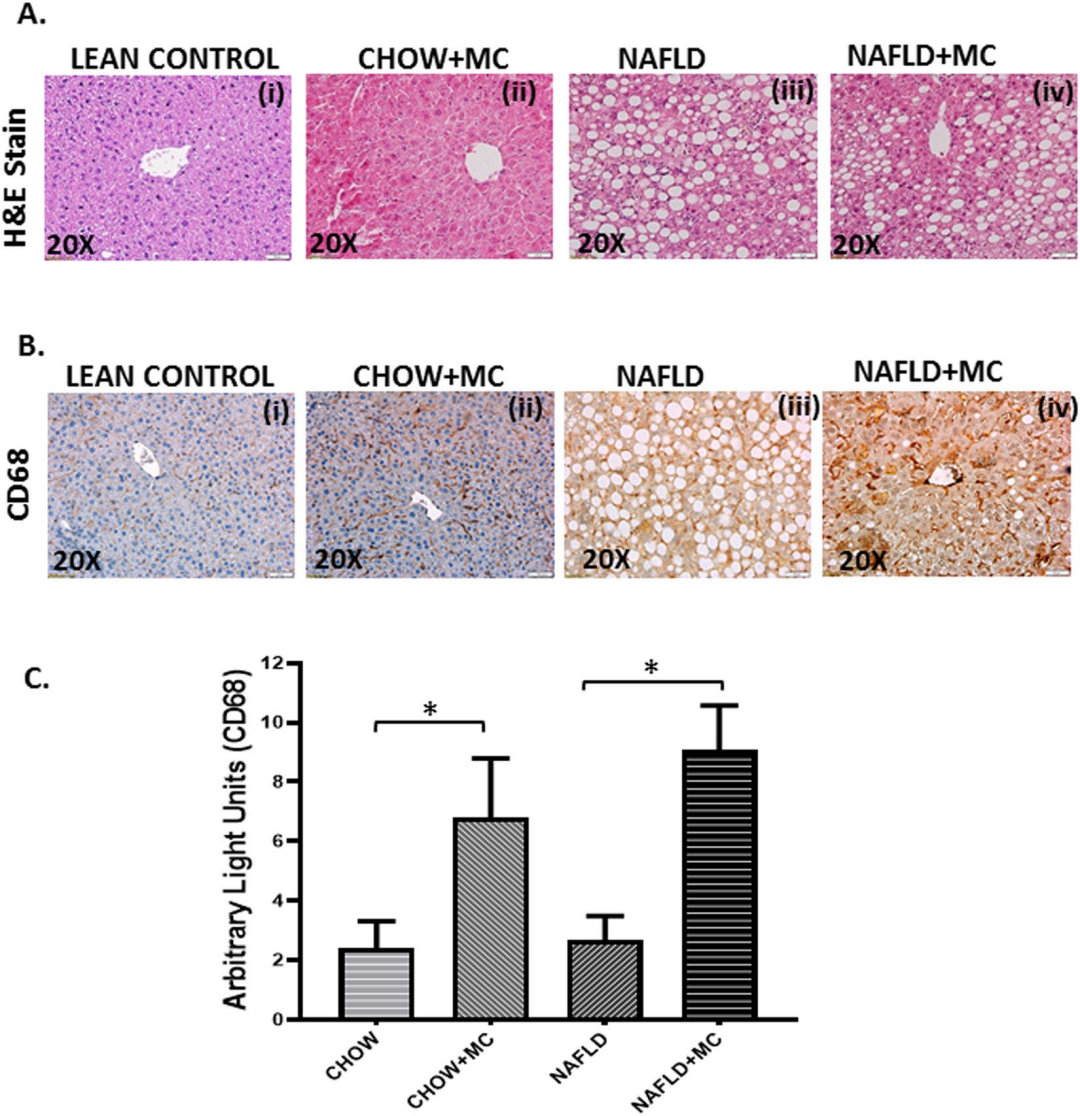
Pathology of microcystin exposure in liver. Formalin-fixed, paraffin-embedded 5 μm liver slices from lean mouse control (also denoted as CHOW) (N = 8), wild type mouse control (NAFLD) (N = 8), wild type mouse group exposed with Microcystin (CHOW + MC) (N = 8) and another NAFLD group of mice exposed with Microcystin (NAFLD + MC) (N = 8) were used for hematoxylin and eosin imaging. (**A**) Representative Hematoxylin and Eosin stained (H&E) images (20×) of liver sections showed NAFLD pathophysiology of control mice group, wild type NAFLD (WT-NAFLD), control group exposed to microcystin (CHOW + MC), and another group of NAFLD mice group exposed to Microcystin. (**B**) (i–iv) Representative Immunohistochemistry images (20×) depicting CD68 immuno-reactivity (**C**) Morphometric analysis of CD68 immunoreactivity (*p < 0.05).

Microcystin is known to primarily affect the liver, being a potential hepatotoxin. To study the effects of microcystin in the liver, we fed the mice with MCD-HFD diet to induce the condition of NAFLD, and then exposed them with microcystin (NAFLD + MC). There was high deposition of fat droplets in the NAFLD liver tissue compared to the lean control mouse. The liver tissue from the NAFLD + MC showed increased inflammation, with infiltration of leukocytes when compared to the chow diet fed mice exposed with Microcystin (Fig. 2Ai–iv). CD68 is known to be the marker for activated Kupffer cells during liver inflammation. To determine whether microcystin had a role in activating the Kupffer cells in the liver, the immunoreactivities of Kupffer cells were analyzed in liver tissue slices (Fig. 2Bi–iv,C). The results showed a significant increase in CD68 immunoreactivity in the CHOW + MC group compared to the lean control mice (CHOW) (*p < 0.05), and simultaneously a significant increase in NAFLD + MC mice compared to the NAFLD only group (*p < 0.05).

### Microcystin exposure in underlying NAFLD worsens gut microbiome changes

**Figure 3:**
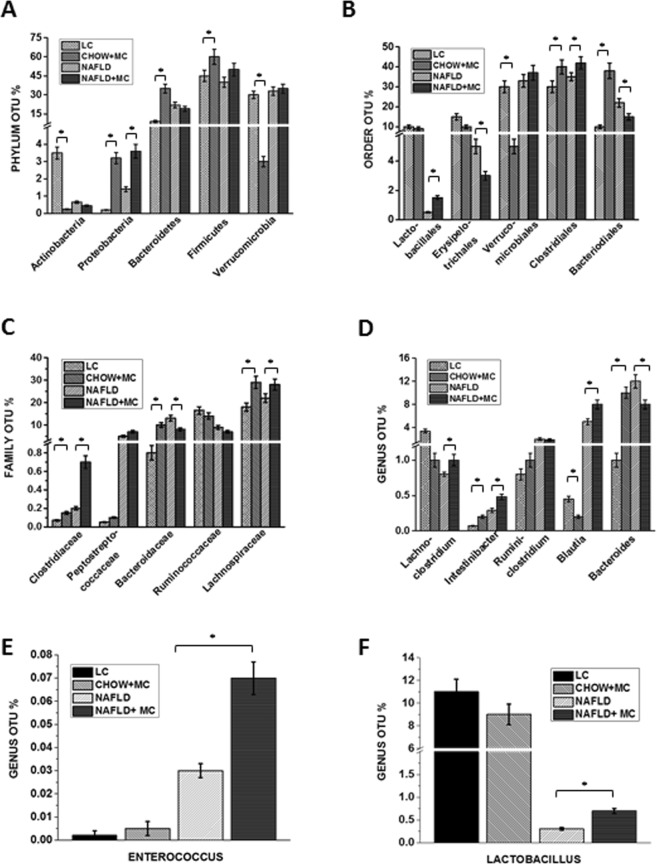
Dysbiosis in NAFLD. Fecal pellets and luminal contents were collected from the animals of each group after sacrifice and microbial analysis was done for the groups Lean Control (LC) (N = 8), CHOW + MC(N = 8), NAFLD(N = 8) and NAFLD + MC(N = 8). (**A**) Microbiome analysis (in %OTU) of the phylum Actinobacteria, Firmicutes, Bacteriodetes, Verrucomicrobia, Proteobacteria (**B**) Microbiome analysis (in %OTU) of the order Bacteriodales, Clostridiales,Verrucomicrobiales, Lactobacillales, Erysipelotrichales (**C**) Microbiome analysis of the family (in %OTU)Bacteriodaceae, Clostridaceae, Lachnospiraceae, Ruminococcaceae, Peptostreptococcaceae. (**D**) Microbiome analysis of the genus (in %OTU)Bacteriodes, Blautia, Lachnoclostridium, Intestinibacter, Ruminiclostridium (**E,F**) Comparison of the lactate producing bacteria from the between Lactobacillus and Enterococcus among the Lean Control, CHOW + MC, NAFLD and NAFLD + MC groups.

NAFLD is accompanied by altered gut microbiota with increases in phylum abundance of Firmicutes and a subsequent decrease in Bacteriodetes^21^. Similar trends are also observed in NAFLD associated inflammatory bowel disease^22^. To study the effects of chronic low dose microcystin exposure in NAFLD, metagenomic analysis of fecal pellets in mice were analyzed. Results showed that NAFLD + MC group had an increase in the phylum Firmicutes compared to the NAFLD only group, and a significant increase in proteobacteria (p < 0.01) followed by a marked decrease in the phylum Bacteroidetes when compared to NAFLD only group (Fig. 3A). Similar trends of a significant increase were observed in the phylum abundance of Firmicutes and proteobacteria of the Chow + MC group, compared to the Lean Control (LC) group (Fig. 3A). There was a significant decrease in the phylum abundance of Verrucomicrobia in the Chow + MC group compared to the Lean Control (p < 0.01). No significant changes were observed in the phylum abundance of Verrucomicrobia in the NAFLD + MC group, when compared to the NAFLD only group (Fig. 3A). The order Clostridiales showed a significant increase in abundance in the NAFLD + MC group when compared to NAFLD only group while there was a significant decrease in the abundance of Bacteriodales in the NAFLD + MC group when compared to NAFLD only group (P < 0.01) (Fig. 3B). The order abundance of Clostridiales also showed a significant increase in the Chow + MC group, compared to the Lean control (LC) group. Verrucomicrobiales showed a significant decrease in the Chow + MC group compared to the Lean Control (LC) group (Fig. 3B). Analysis of the abundance of family showed a significant increase in Clostridiaceae in NAFLD + MC groups when compared to NAFLD only group (p < 0.01) followed by increase in Lachnospiraceae, an observation consistent with both IBD and NAFLD (Fig. 3C). Similar trends of the significant increase in the family of Clostridiaceae and Lachnospiraceae (p < 0.01) in Chow + MC group compared to the lean control (LC) group were also observed (Fig. 3C). At the genus level, abundance of Bacteriodes decreased significantly in the NAFLD + MC group when compared to NAFLD only group (p < 0.01) while Blautia and Intestinibacter abundance increased significantly in the NAFLD + MC group when compared to NAFLD only group (p < 0.05)(Fig. 3D). Increase in the Intestinibacter genus abundance was observed in the Chow + MC group, compared to the Lean Control (LC) group (Fig. 3D). Lactobacillus and Enterococcus genus that are widely known as lactate producing bacteria increased significantly in NAFLD + MC group when compared to NAFLD only group (p < 0.05)(Fig. 3E,F)^23^. The data is consistent with human NAFLD and intestinal comorbidities severity since the increased level of the genus Intestinibacter, the family Lachnospiraceae, the genus Escherichia, Shigella, and the family Enterobacteriaceae may be a primary contributor to NAFLD progression^24^.

### Microcystin exposure and resultant worsening dysbiosis in NAFLD is positively associated with altered intestinal pathology

**Figure 4:**
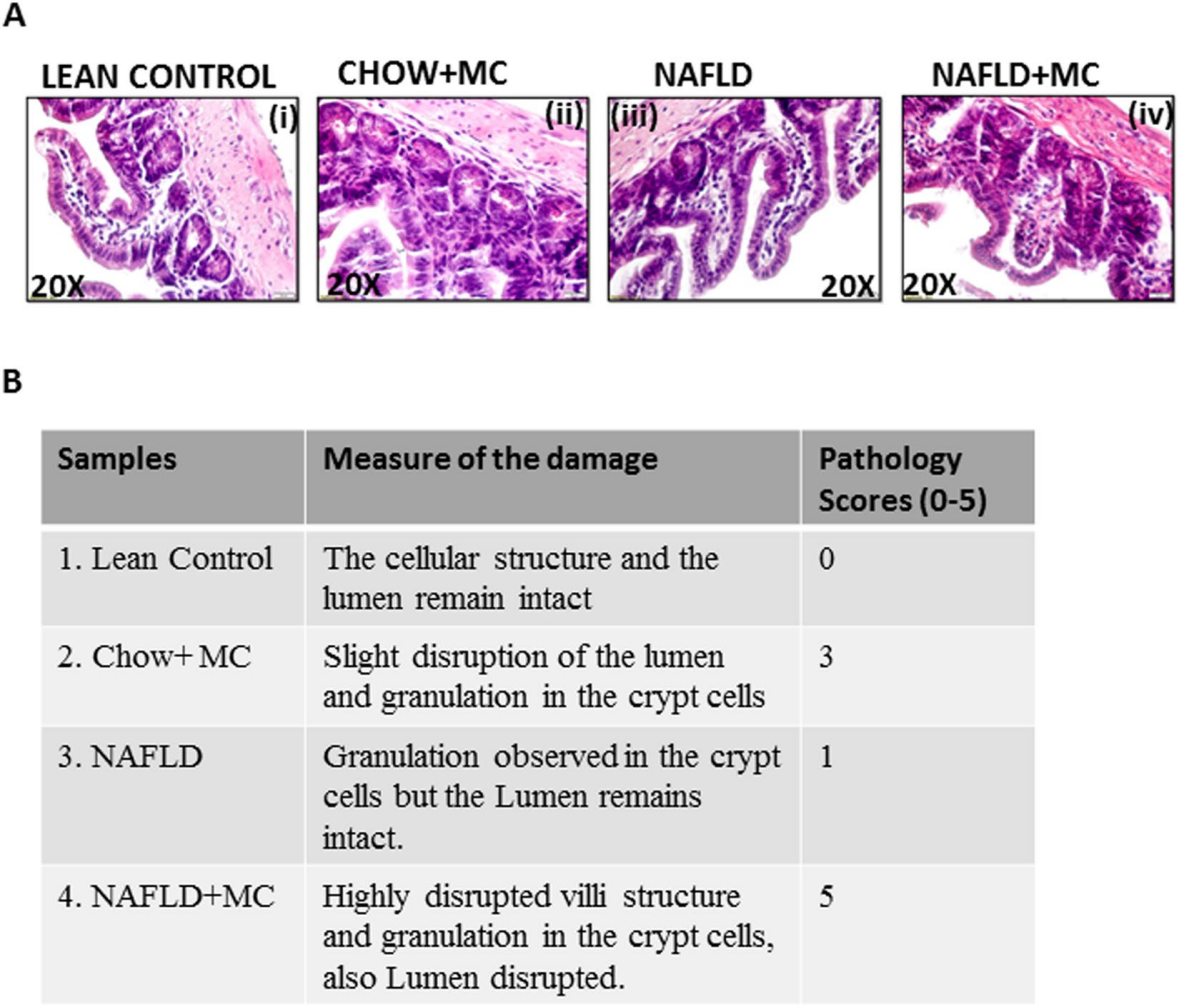
(**A**) Intestinal Pathology in NAFLD associated Dysbiosis and on Microcystin exposure. Formalin-fixed, paraffin-embedded 5 μm intestinal slices from lean mouse control (N = 8), wild type mouse control (NAFLD) (N = 8), wild type mouse group exposed with Microcystin (CHOW + MC) (N = 8) and another NAFLD group of mice exposed with Microcystin (NAFLD + MC) (N = 8) were used for hematoxylin and eosin imaging. Representative Hematoxylin and Eosin stained(H&E) images (20×) of intestinal sections showed NAFLD pathophysiology of control mice group, wild type NAFLD (WT-NAFLD), control group exposed to microcystin (CHOW + MC), and another group of NAFLD mice group exposed to Microcystin. (**B**) Pathological Scores in NAFLD associated damage on Microcystin exposure. The pathological scores were assigned arbitrarily according to the extent of damage, disruption in the lumen and granulation in crypt cells caused in the small intestine.

Intestinal pathology in inflammatory bowel disease assumes a marked signature of epithelial cell loss, disruption of villous structures, crypt abscess and granulation^25^. To study the effect of microcystin exposure and the resultant worsened dysbiosis in NAFLD, a detailed histopathological examination was carried out. Results showed that NAFLD + MC had highly disrupted villi structure, increased granulation in the crypt cells, crypt cell abscess, erosion of epithelial cells and disrupted lumen when compared to lean control, Chow + MC group and NAFLD only group respectively (Fig. 4A(i–iv),B). NAFLD + MC group showed non-parallel crypts, variable crypt diameters, bifurcation and branched crypts, consistent with significantly higher inflammation scores. Infiltrating leukocytes were fairly common in the disruptive villi and in the crypts of NAFLD + MC group when compared to lean, Chow + MC and NAFLD groups respectively. The results suggested that microcystin exposure in NAFLD that had consistent dysbiosis were positively associated with moderate to severe inflammatory phenotype in the intestines.

### Microcystin exposure and resultant worsening dysbiosis in NAFLD is positively associated with increased levels of infiltrating leukocytes and NLRP3 inflammasome activation

**Figure 5:**
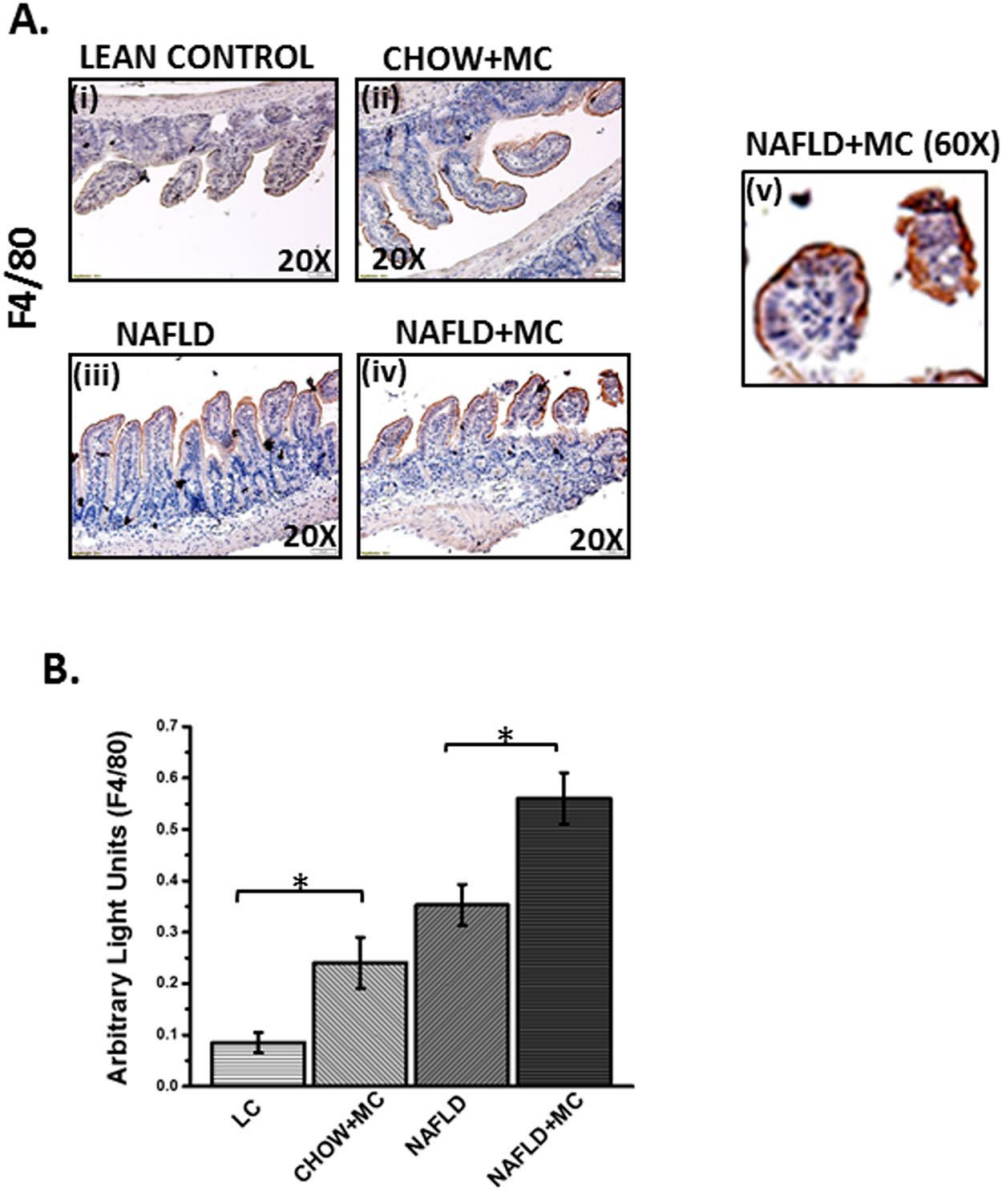
Formalin-fixed, paraffin-embedded 5 µm intestinal slices from lean mouse control (No. of mice per group, N = 8), wild type mouse control (NAFLD) (N = 8), wild type mouse group exposed with Microcystin (CHOW + MC) (N = 8) and another NAFLD group of mice exposed with Microcystin (NAFLD + MC) (N = 8)were used for immunohistochemistry imaging. **(A)** (i–iv) Representative Immunohistochemistry images (20X) and (v) higher magnification image (60X) of NAFLD + MC group depicting F4/80 immunoreactivity. (**B**) Morphometric analysis of F4/80 immunoreactivity (*p < 0.05).

**Figure 6:**
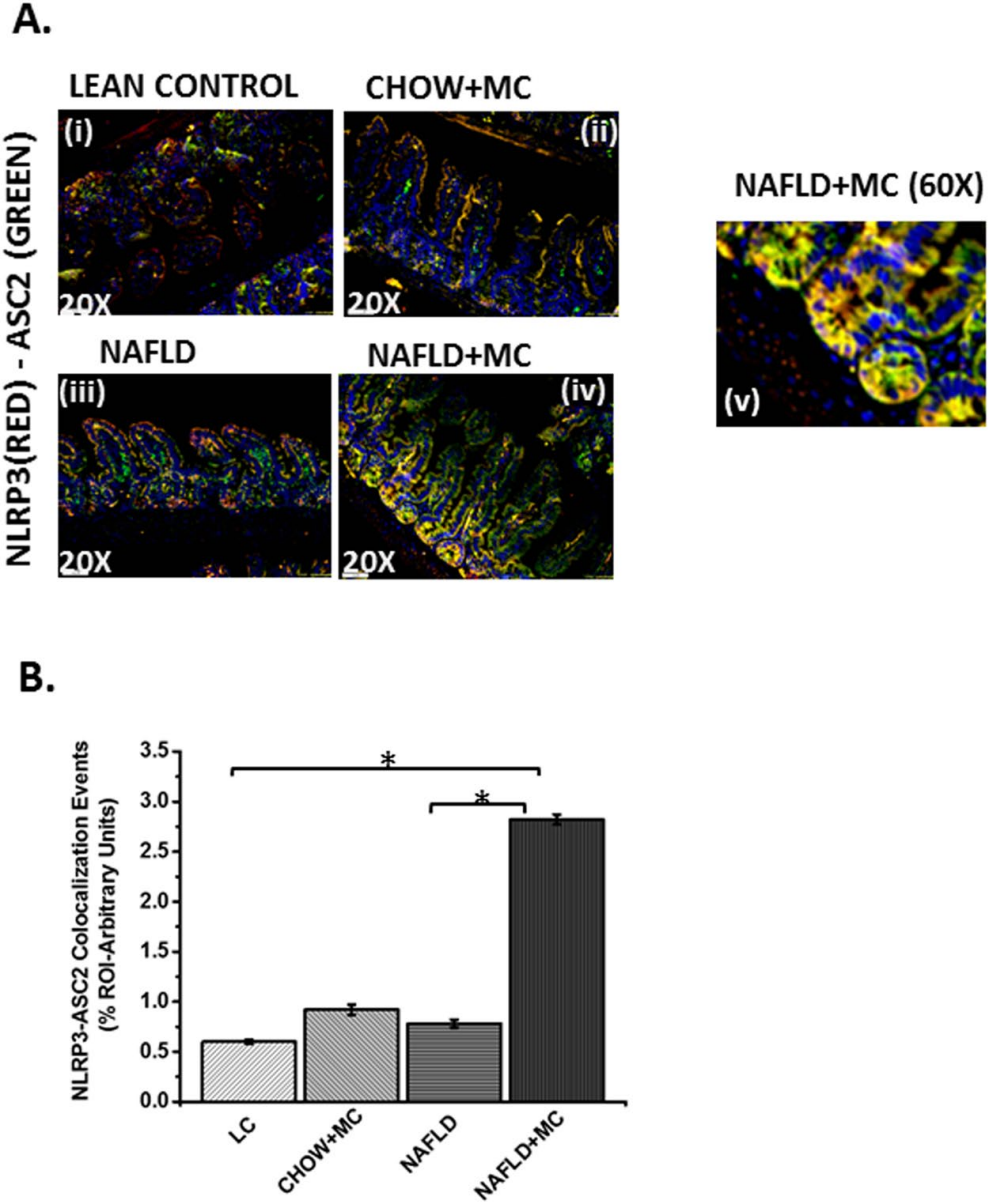
Inflammasome Formation: Formalin-fixed, paraffin-embedded 5 µm intestinal slices from lean mouse control (No. of mice per group, N = 8), wild type mouse control (NAFLD) (N = 8), wild type mouse group exposed with Microcystin (CHOW + MC) (N = 8) and another NAFLD group of mice exposed with Microcystin (NAFLD + MC) (N = 8) were used for immunofluorescence imaging. (**A**) (i–iv) Immunofluorescence images in 20X depicting NLRP3 (red) and ASC2 (green) colocalization, counterstained with DAPI (blue) in LC, CHOW + MC, NAFLD and NAFLD + MC group of mice intestine. (v) A higher magnification image was taken at 60 X magnification for the NAFLD + MC group. (**B**) Morphometric analysis of NLRP3-ASC2 colocalization immunoreactivity. Y-axis shows % positive immunoreactive area (% ROI) (n = 5, analysis from five separate microscopic fields) (*p < 0.05).

Intestinal inflammation in moderate to severe pathology associated with inflammatory bowel disease phenotypes show consistent infiltration of leukocytes and NLRP3-inflammasome activation^26^. To study the above-mentioned effects and their association with microcystin exposure, immunohistochemistry of stained slides were analyzed for pan macrophage marker F4/80. Results showed that NAFLD + MC group had significantly increased F4/80 immunoreactivity when compared to lean control, Chow + MC and NAFLD only groups (Fig. 5A,B) (P < 0.01). The extent of staining for F4/80 and localizations were shown by a higher magnification image (Fig. 5Av). NLRP3 inflammasome activation is a significant event in intestinal inflammation and is often associated with downstream IL1β release and pyroptosis^27^. To show the effect of microcystin exposure onNLRP3 inflammasome activation, immunoreactivities were analyzed for NLRP3 and ASCII. Interestingly, activation of the inflammasome is marked by their colocalization thus forming a protein complex^27^. Results showed that colocalization events of NLRP3 and ASCII increased significantly in NAFLD + MC group when compared to lean, Chow + MC and NAFLD groups (Fig. 6A(i–iv),B). A representative higher magnification image showing the increased colocalizations of NLRP3 and ASC II is shown in Fig. 6A-v. The above results showed that the observations were consistent with the histopathology of the affected intestines shown in Fig. 4. Interestingly the colocalizations were highly visible in the crypts that had increased granulation and abscess.

### Microcystin exposure in NAFLD is positively associated with alteration of tight junction protein levels and gut leaching leading to systemic low level endotoxemia

**Figure 7:**
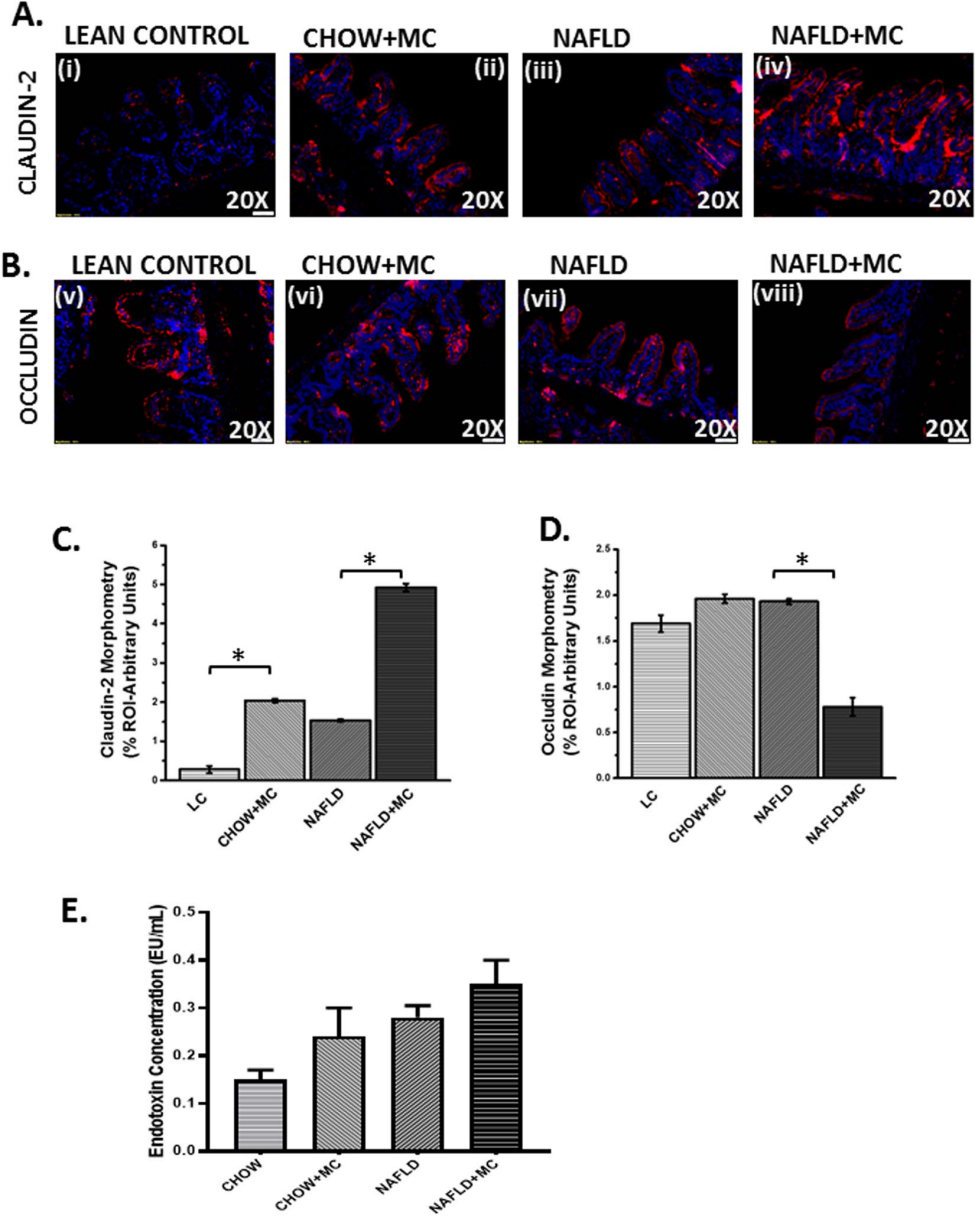
Gut Leaching and Systemic Endotoxemia: Formalin-fixed, paraffin-embedded 5 μm intestinal slices from lean mouse control (No. of mice per group, N = 8), wild type mouse control (NAFLD) (N = 8), wild type mouse group exposed with Microcystin (CHOW + MC) (N = 8) and another NAFLD group of mice exposed with Microcystin (NAFLD + MC) (N = 8) were used for immunofluorescence imaging. (**A**) Immunofluorescence images depicting Claudin-2 (red) (i–iv) and (**B**) Occludin (red) (v–viii), counterstained with DAPI (blue) in LC, CHOW + MC, NAFLD and NAFLD + MC group of mice intestine. Images were taken at 20X magnification. (**C**) Morphometric analysis of Claudin-2 immunoreactivity. Y-axis shows % positive immunoreactive area (% ROI) (n = 5, analysis from five separate microscopic fields) (*p < 0.05) (**D**) Morphometric analysis of Occludin immunoreactivity. Y-axis shows % positive immunoreactive area (% ROI) (n = 5, analysis from five separate microscopic fields) (0.01 < *p < 0.05). (**E**) Systemic endotoxemia was measured by Chromogenic LAL assay in serum of LC (CHOW), CHOW + MC, NAFLD and NAFLD + MC group of mice.

We and others have shown previously that alteration of gut dysbiosis leads to gut leaching and systemic endotoxemia^28,29^. The studies are also consistent with NAFLD and IBD where similar results are reported. To show that microcystin exposure in an underlying NAFLD could alter tight junction proteins claudin-2 and occluding, mice intestinal tissue slices were analyzed for immunoreactivities of the above-mentioned proteins. Results showed that the levels of claudin-2 were significantly increased in NAFLD + MC group when compared to lean control, Chow + MC and NAFLD groups (Fig. 7Ai–iv,C (P < 0.05)). Occludin levels were significantly decreased in the NAFLD + MC group when compared to lean control, Chow + MC and NAFLD groups (P < 0.05)(Fig. 7Bv–viii,D). Interestingly, microcystin exposure to lean control mice also increased claudin-2 levels significantly suggesting MC exposure can lead to altered tight junction proteins independently however the conditioned is worsened in an underlying NAFLD. We and others have shown that microbial dysbiosis leads to gut leaching and low systemic endotoxemia, a condition very much associated with intestinal inflammation^29^. Results showed that there was a marked increase in systemic endotoxin levels in NAFLD + MC group when compared to Chow + MC and NAFLD groups (Fig. 7E) suggesting the dysbiosis, inflammatory phenotypes and altered tight junction proteins in NAFLD + MC groups were strongly associated with systemic endotoxemia.

### Microcystin exposure in an underlying NAFLD exacerbated oxidative stress, radical formation and was associated with increased activation of NADPH Oxidase 2 (NOX-2)

**Figure 8:**
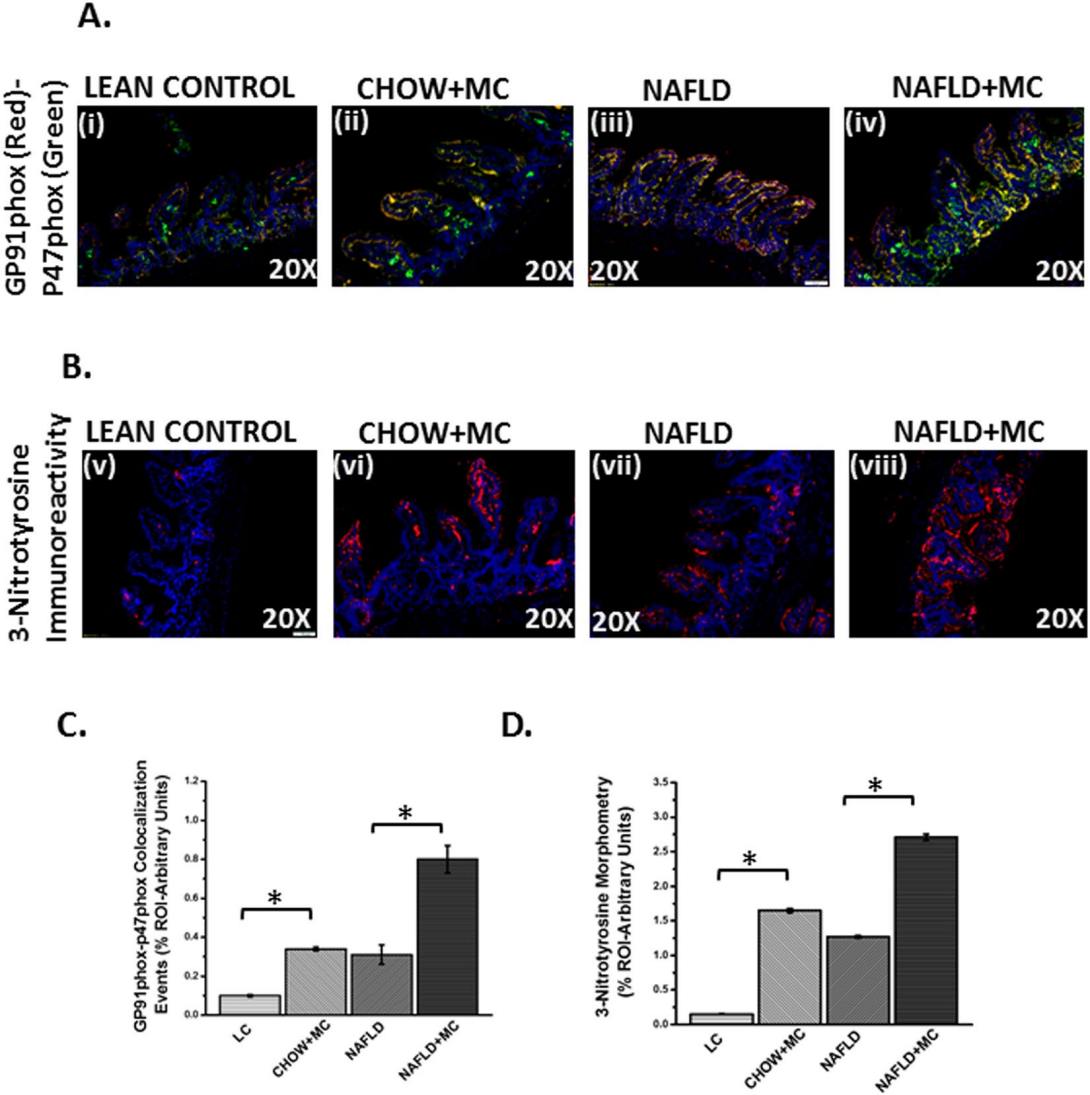
Oxidative Stress and activation of NOX2: Formalin-fixed, paraffin-embedded 5 µm intestinal slices from lean mouse control (No. of mice per group, N = 8), wild type mouse control (NAFLD) (N = 8), wild type mouse group exposed with Microcystin (CHOW + MC) (N = 8) and another NAFLD group of mice exposed with Microcystin (NAFLD + MC) (N = 8) were used for immunofluorescence imaging. (**A**) (i–iv) Immunofluorescence images depicting gp91 phox (red) and p47 phox (green) colocalization, counterstained with DAPI (blue) in LC, CHOW + MC, NAFLD and NAFLD + MC group of mice intestine. Images were taken at 20X magnification. (**B**) (v–viii) Immunofluorescence images depicting 3-Nitrotyrosine (red), counterstained with DAPI (blue) in LC, CHOW + MC, NAFLD and NAFLD + MC group of mice intestine. Images were taken at 20X magnification. (**C**) Morphometric analysis of gp91 phox-p47phox colocalized immunoreactivity. Y-axis shows % positive immunoreactive area (% ROI) (n = 5, analysis from five separate microscopic fields) (0.01 < *p < 0.05). (**D**) Morphometric analysis of 3-Nitrotyrosine immunoreactivity. Y-axis shows % positive immunoreactive area (% ROI) (n = 5, analysis from five separate microscopic fields) (*p < 0.05).

Oxidative stress is central to environmental exposure of toxins following their secondary metabolism and affects tissue behavior^13,30,31^. Interestingly we have shown before that microbiome changes can cause oxidative stress though the exact mechanism remained unclear^29^. Based on the strong results of an inflammatory pathology in the intestine, we chose to study the mechanisms of oxidative stress following microcystin induced dysbiosis in an underlying NAFLD intestinal tissue. Results showed that there was a significant increase inNOX-2 membrane assembly and activation as shown by the colocalization of the GP91 phox and P47 phox in the intestine when compared to lean control, Chow + MC and NAFLD groups (p < 0.05)(Fig. 8Ai–iv), C). Tyrosine radical formation and subsequent tyrosine nitration, a marker of oxidative stress was significantly elevated in the NAFLD + MC group when compared to lean control, Chow + MC and NAFLD group respectively (P < 0.05)(Fig. 8Bv–viii,D)^32^. The results suggested that microcystin exposure and gut dysbiosis correlated well with increased oxidative stress primarily through intestinal NOX-2 activation and tyrosine nitration. It is worth mentioning that oxidative stress enzymes known to increase radical species such as xanthine oxidase or myeloperoxidase levels were unchanged in these treatment groups (data not shown) suggesting that NOX2-induced oxidative insult may be prime for downstream inflammatory processes and disease phenotypes or vice versa which is unexplained at this point.

### NOX2 phenotype is downstream of associated dysbiosis and causes inflammasome activation and proinflammatory IL1b release typical of the NLRP3-ASC-2 axis

**Figure 9:**
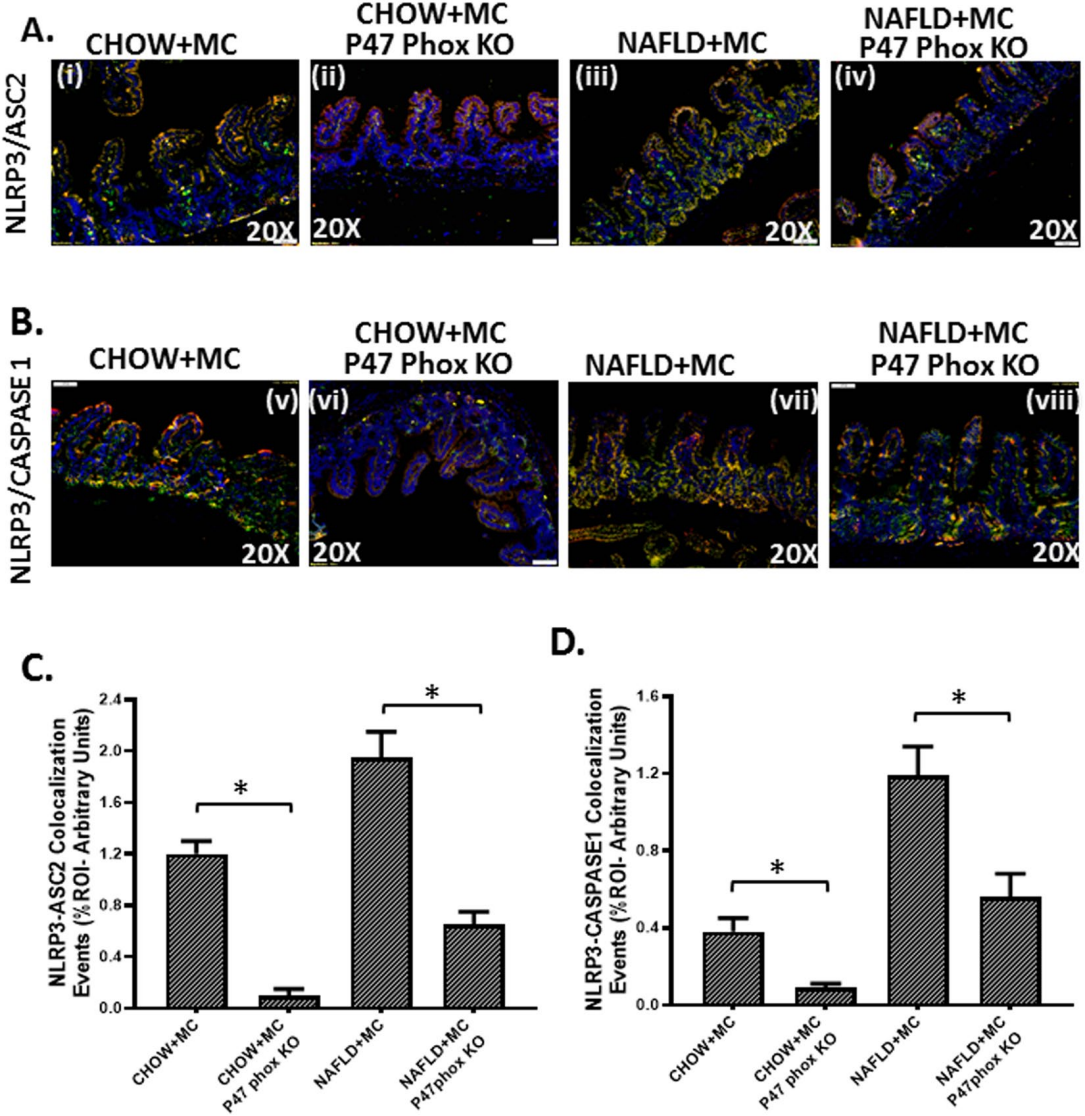
NOX2 activation by Microcystin is prime to intestinal inflammasome activation: Formalin-fixed, paraffin-embedded 5 μm intestinal slices from wild type mouse group exposed with Microcystin (CHOW + MC) (No. of mice per group, N = 8), CHOW + MC group with P47 phox gene knock out (*P47phox KO*) (N = 8), NAFLD wild type group of mice exposed with Microcystin (NAFLD + MC) (N = 8) and another NAFLD + MC group with P47 phox gene knock out (*P47phox KO*) (N = 8) were used for immunofluorescence and immunohistochemistry imaging. (**A)** (i–iv) Immunofluorescence images depicting NLRP3 (red) and ASC2 (green) colocalization, counterstained with DAPI (blue) in CHOW + MC, CHOW + MC(*P47phoxKO*), NAFLD + M C, NAFLD + MC(*P47phox KO*) group of mice intestine. Images were taken at 20X magnification. (**B**) (v–viii) Immunofluorescence images depicting NLRP3 (red) and Caspase-1 (green) colocalization, counterstained with DAPI (blue) in CHOW + MC,CHOW + MC (*P47phoxKO*), NAFLD + MC, NAFLD + MC(*P47phox KO*) group of mice intestine. Images were taken at 20X magnification. (**C)** Morphometric analysis of NLRP3-ASC2 colocalized immunoreactivity. Y-axis shows % positive immunoreactive area (% ROI) (n = 5, analysis from five separate microscopic fields) (*p < 0.05). **(D)** Morphometric analysis of NLRP3-Caspase-1 colocalized immunoreactivity. Y-axis shows % positive immunoreactive area (% ROI) (n = 5, analysis from five separate microscopic fields) (0.01 < *p < 0.05).

**Figure 10:**
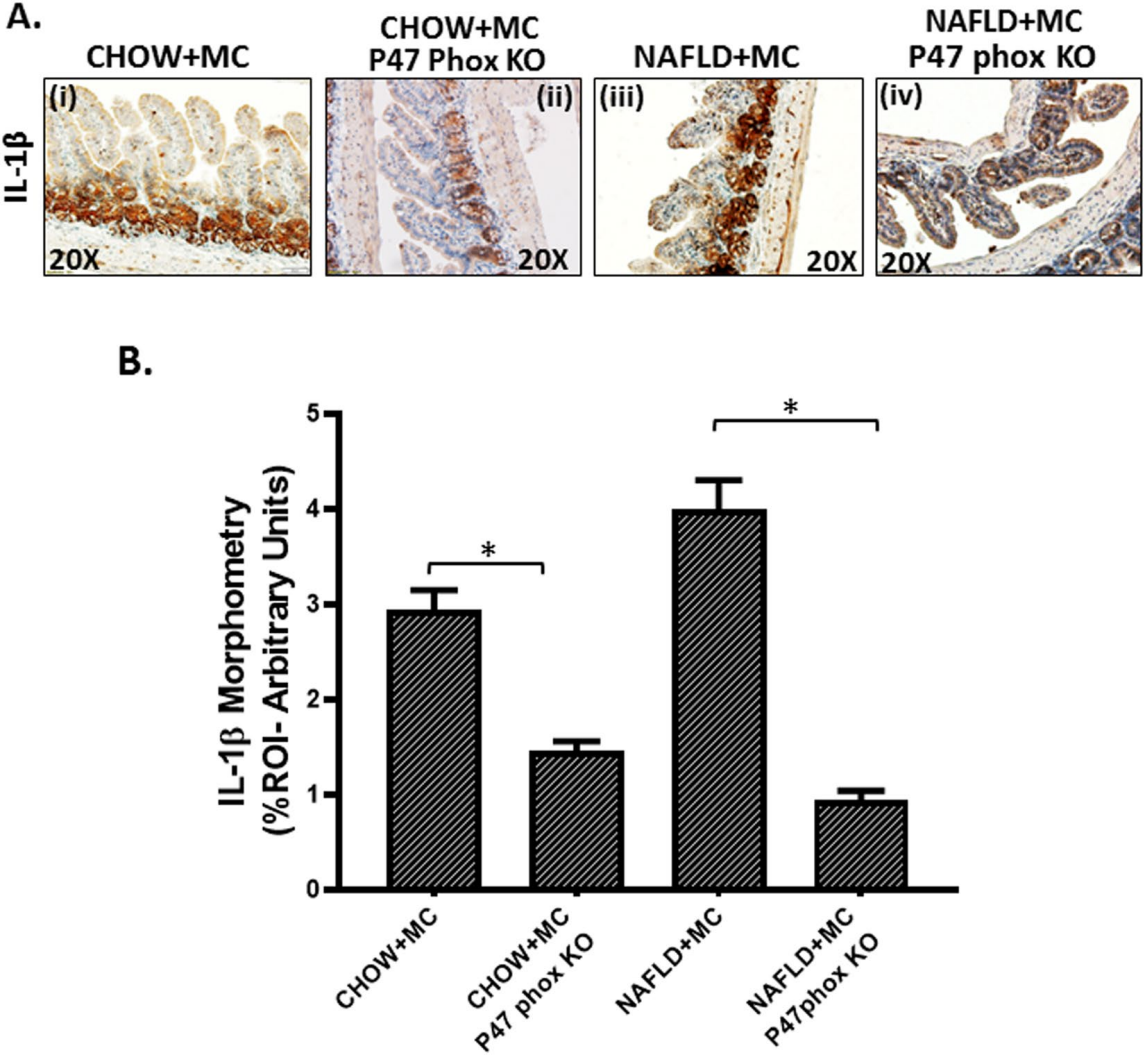
Formalin-fixed, paraffin-embedded 5 µm intestinal slices from wild type mouse group exposed with Microcystin (CHOW + MC) (No. of mice per group, N = 8), CHOW + MC group with P47 phox gene knock out (*P47phox KO*) (N = 8), NAFLD group of mice exposed with Microcystin (NAFLD + MC) (N = 8) and another NAFLD + MC group with P47 phox gene knock out (*P47phox KO*) (N = 3) were used for immunohistochemistry imaging. (**A**) (i–iv) Immunohistochemistry images depicting IL-1β immunoreactivity in CHOW + MC, CHOW + MC (*P47phox KO)*, NAFLD + MC, NAFLD + MC(*P47phox KO*) group of mice intestine. Images were taken at 20X magnification. **(B)**Morphometric analysis of IL-1β immunoreactivity. Y-axis shows % positive immunoreactive area (% ROI) analysis from n = 5, five separate microscopic fields) (*p < 0.05).

To study the role of NOX-2 in the inflammatory changes in the NAFLD intestine following microcystin exposure we used mice that have the genetic deletion of the cytosolic component of NOX-2 (henceforth referred to as P47 phox knockout mice). The p47 phox KO group was treated identically as the CHOW + MC and the NAFLD + MC groups. Results showed that CHOW + MC (P47 phox KO) and NAFLD + MC (P47 phox KO) mice showed a significant decrease in colocalization of NLRP3/ASC II when compared to CHOW + MC and NAFLD + MC groups respectively (Fig. 9Ai–iv,C). Inflammasome activation is also dependent on the binding of Caspase 1 with NLRP3 to form a stable inflammasome complex^33^. NLRP3 and Caspase-1 colocalization events were significantly decreased in the P47 phox KO group when compared to CHOW + MC and NAFLD + MC groups suggesting a decreased NLRP3 inflammasome activation (p < 0.05)(Fig. 9Bv–viii,D). Inflammasome activation is followed by a release of IL-1β for the pathway to proceed downstream^26^. IL1β protein levels as measured by immunohistochemistry decreased significantly in the p47 phox KO group when compared to CHOW + MC and NAFLD + MC groups suggesting that NOX-2 was required for the inflammasome activation and IL1β release in the diseased intestine (Fig. 10Ai–iv,B) (P < 0.05). Interestingly, the IL1β was localized in the same region as the NOX-2 suggesting the histopathological sites of the events are likely the same.

### NOX2 is essential for alteration of gut adhesion via regulating the protein levels of tight junctions, release of HMGB1 and serum endotoxemia

**Figure 11:**
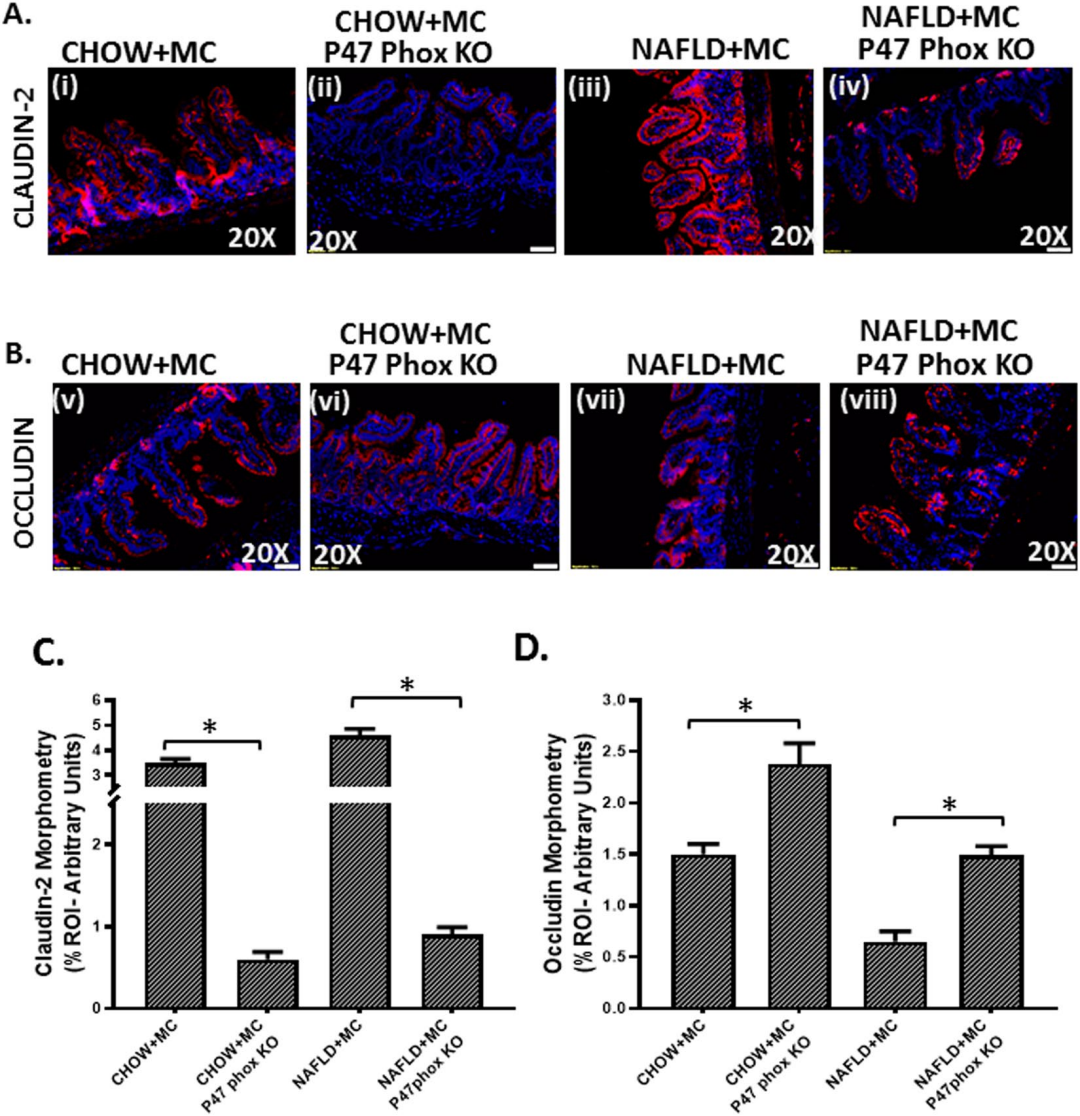
NOX2 activation by Microcystin is prime to alteration of gut barrier integrity: Formalin-fixed, paraffin-embedded 5 μm intestinal slices from wild type mouse group exposed with Microcystin (CHOW + MC) (No. of mice per group, N = 8), CHOW + MC group with P47 phox gene knock out (*P47phox KO*) (N = 8), NAFLD wild type group of mice exposed with Microcystin (NAFLD + MC) (N = 8) and another NAFLD + MC group with P47 phox gene knock out (*P47phox KO*) (N = 8) were used for immunofluorescence and immunohistochemistry imaging. (**A**) (i–iv) Immunofluorescence images depicting Claudin-2 (red), counterstained with DAPI (blue) in CHOW + MC,CHOW + MC (*P47phox KO*),NAFLD + MC, NAFLD + MC(*P47phox KO*) group of mice intestine. Images were taken at 20X magnification. (**B**) (v-viii)Immunofluorescence images depicting Occludin (red), counterstained with DAPI (blue) in CHOW + MC, CHOW + MC (*P47phox KO*), NAFLD + MC, NAFLD + MC(*P47phox KO*) group of mice intestine. Images were taken at 20X magnification. **(C)** Morphometric analysis of Claudin-2 immunoreactivity. Y-axis shows % positive immunoreactive area (% ROI) (n = 5, analysis from five separate microscopic fields) (*p < 0.05). **(D)** Morphometric analysis of Occludin immunoreactivity. Y-axis shows % positive immunoreactive area (% ROI) (n = 5, analysis from five separate microscopic fields) (0.01 < *p < 0.05).

**Figure 12:**
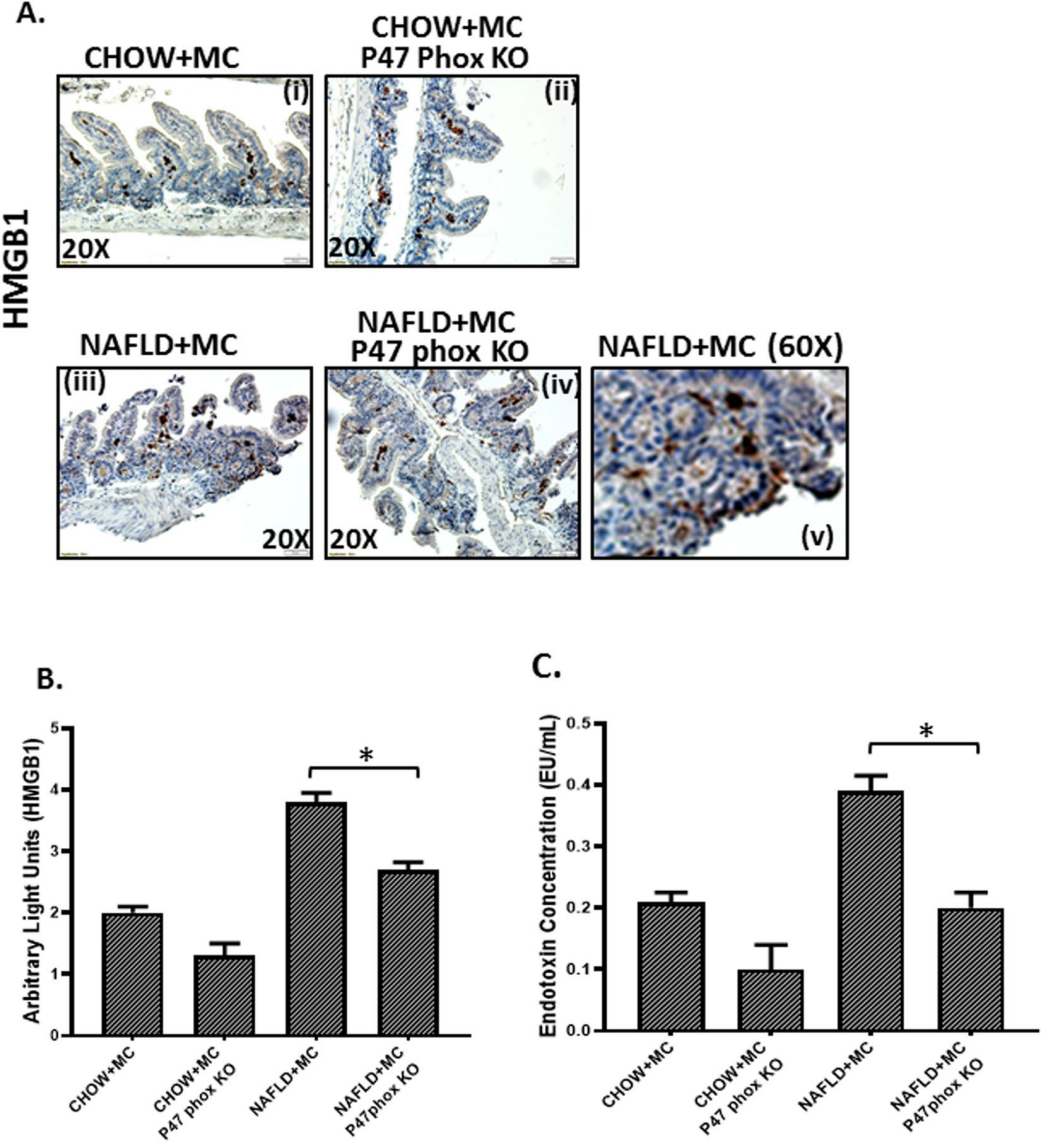
Formalin-fixed, paraffin-embedded 5 μm intestinal slices from wild type mouse group exposed with Microcystin (CHOW + MC) (No. of mice per group, N = 8), CHOW + MC group with P47 phox gene knock out (*P47phox KO*) (N = 8), NAFLD wild type group of mice exposed with Microcystin (NAFLD + MC) (N = 8) and another NAFLD + MC group with P47 phox gene knock out (*P47phox KO*) (N = 8) were used for immunohistochemistry imaging. (**A**) (i–iv) Immunohistochemistry images depicting HMGB1 immunoreactivity in CHOW + MC, CHOW + MC (*P47phox KO*), NAFLD + MC, NAFLD + MC (*P47phox KO*) group of mice intestine. Images were taken at 20X magnification and **(v)** higher magnification image (60X) of NAFLD + MC group depicting HMGB1 immunoreactivity. **(B**) Morphometric analysis of HMGB1 immunoreactivity. Y-axis shows % positive immunoreactive area (% ROI) (n = 5, analysis from five separate microscopic fields). **(C)** Systemic endotoxemia was measured by Chromogenic LAL assay in serum of CHOW + MC, CHOW + MC (*P47phox KO*), NAFLD + MC and NAFLD + MC (*P47phox KO*) group of mice (*p < 0.05).

Results showed that use of P47 phox knockout mice for both the CHOW + MC and NAFLD + MC groups showed a significant decrease in Claudin-2 protein levels when compared to CHOW + MC and NAFLD + MC groups respectively (P < 0.05) Fig. 11Ai–iv,C). The levels of another tight junction protein Occludin increased significantly in the CHOW + MC (P47 phox KO) group when compared to CHOW + MC group, and also there was a significant increase in NAFLD + MC (P47 phox KO) group when compared to NAFLD + MC group suggesting that NOX2 was important for the alterations of these proteins crucial for maintaining gut barrier integrity (p < 0.05)(Fig. 11Bv–viii,D). P47phox KO mice also had decreased protein levels of HMGB1 (Fig. 12Ai–iv,B), a damaged associated molecular protein known to cause inflammatory pathology downstream of NLRP3 inflammasome activation when compared to Chow + MC and NAFLD + MC groups respectively (P < 0.05). Serum endotoxin levels also showed a decreased trend in p47 phox KO group when compared to CHOW + MC and NAFLD + MC groups (Fig. 12C). A representative higher magnification image of NAFLD + MC group suggests that higher HMGB1 was primarily localized in the crypt cells that showed a parallel cell damage and leukocyte infiltration (Fig. 12Ai–iv).

### Microcystin exposure in intestinal epithelial cells causes the generation of peroxynitrite species following NOX-2 activation in the presence of nitric oxide and causes inflammatory phenotype

**Figure 13:**
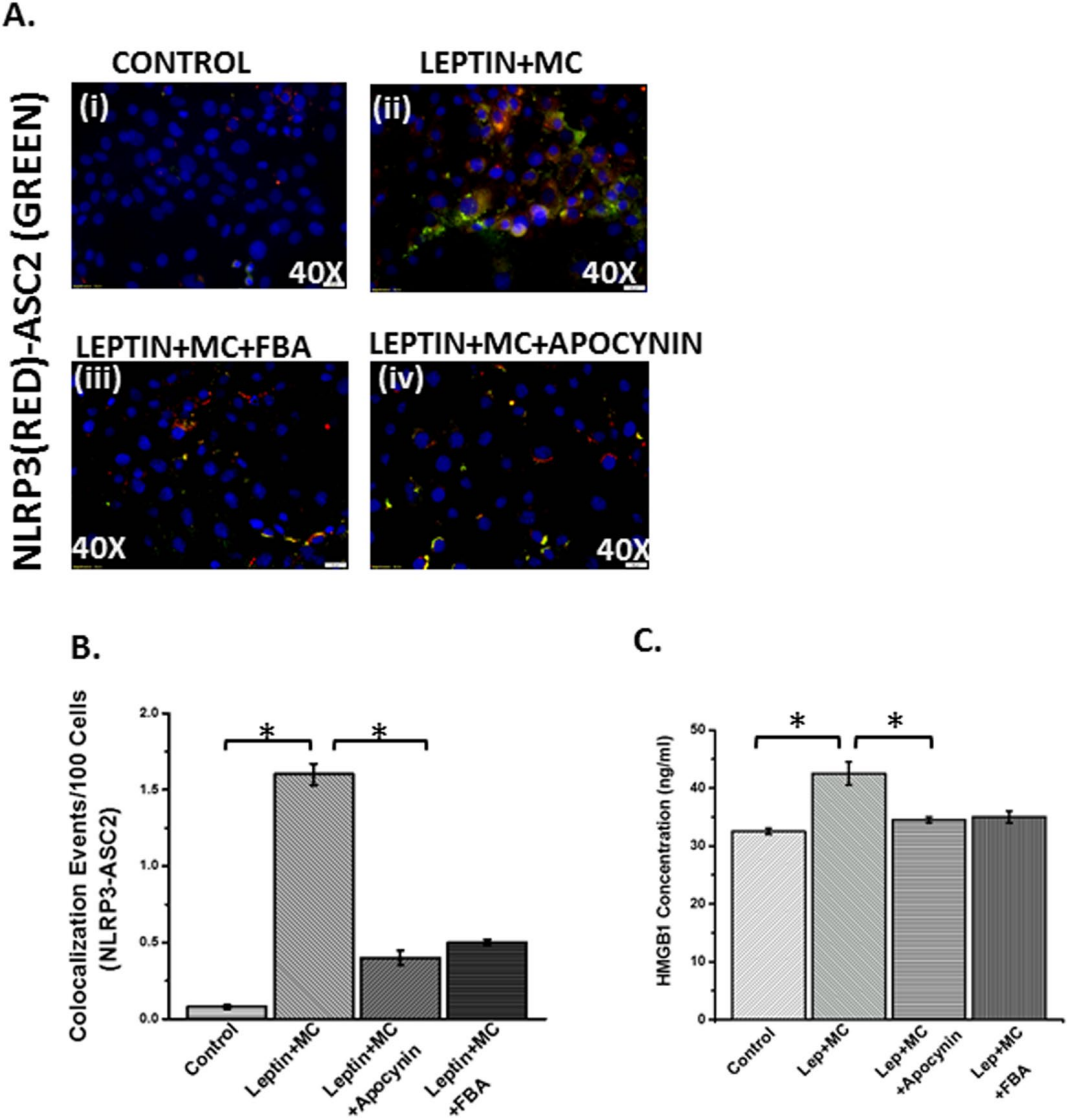
NOX2 induced peroxynitrite primes inflammasome activation in intestinal epithelial cells: Intestinal epithelial (IEC-6) cell line was used as control (CONTROL), NAFLD was induced with leptin in another group which was exposed to Microcystin (LEPTIN + MC), and another group of cells exposed to leptin and microcystin was blocked by Apocynin and FBA (LEPTIN + MC + APOCYNIN) (LEPTIN + MC + FBA). The cell groups were used forimmunofluorescence imaging. (**A**) (i–iv) Immunofluorescence images depicting NLRP3 (red) and ASC2 (green) colocalization, counterstained with DAPI (blue) in CONTROL, LEPTIN + MC, LEPTIN + MC + APOCYNIN, LEPTIN + MC + FBA group of IEC-6 cells. Images were taken at 40X magnification. (**B**) Morphometric analysis of NLRP3-ASC2 colocalized immunoreactivity. Y-axis shows % positive immunoreactive area (% ROI) (n = 5, analysis from five separate microscopic fields) (0.01 < *p < 0.05). **(C)** HMGB1 adducts were measured by ELISA in IEC-6 cell supernatant of CONTROL, LEPTIN + MC, LEPTIN + MC + APOCYNIN, LEPTIN + MC + FBA groups (0.01 < *p < 0.05).

**Figure 14:**
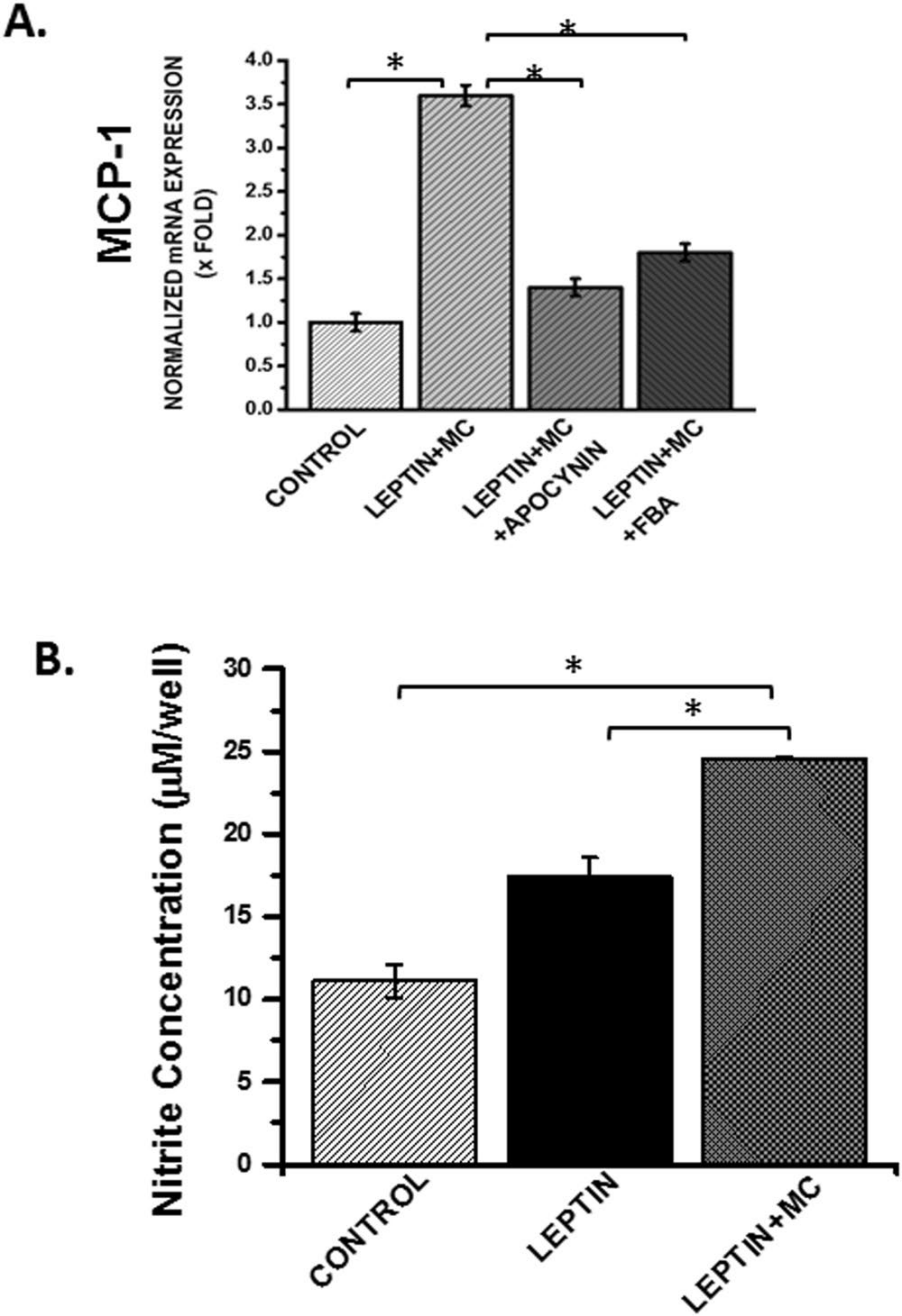
(**A**) mRNA expression of MCP-1 in CONTROL, LEPTIN + MC, LEPTIN + MC + APOCYNIN, LEPTIN + MC + FBA group of IEC-6 cells was performed using qRTPCR. mRNA expression is represented as fold change of NAFLD on Y-axis (0.01 < *p < 0.05). (**B**) Nitric oxide release was detected by the Griess Reagent system using Intestinal Epithelial cells (IEC-6) cell supernatants for the groups CONTROL, LEPTIN, LEPTIN + MC, to study the concentration of nitrite formed per well. (n = 3, analysis from three samples) (*p < 0.05).

We and others have shown that NOX2 activation inthe intestine in an underlying NAFLD is followed by peroxynitrite generation, which subsequently acts to target various cellular signaling processes leading to inflammation^32^. In this study we modelled a cellular system where leptin was used to mimic a cellular NAFLD microenvironment since the disease is accompanied by leptin resistance^34^. This is also supported by the fact that leptin is proinflammatory and usually acts via peroxynitrite to target inflammation^32^. Results showed that microcystin exposure significantly increased NLRP3 inflammasome activation when compared to cell only controls (only leptin controls showed similar result) while incubation with peroxynitrite scavenger phenyl boronic acid (FBA) or NOX2 inhibitor apocynin significantly decreased the activation (Fig. 13Ai–iv,B) (P < 0.05). Release of HMGB1 was also decreased significantly in the FBA and Apocynin groups when compared to Lep + MC group (Fig. 13C) suggesting that NOX2 mediated peroxynitrite generation is crucial for NLRP3-inflammasome activation and DAMP release in these cells. Monocyte chemoattractant protein-1 (MCP-1) is responsible for infiltration of leukocytes at the site of tissue injury^35^. MCP-1 levels were significantly decreased in the epithelial cells treated with both apocynin and FBA suggesting that leukocyte infiltration was also influenced by NOX-2 mediated peroxynitrite generation (Fig. 14A) (P < 0.05).

### Nitric oxide is crucial for NOX2-induced peroxynitrite generation and NLRP3 inflammasome activation

**Figure 15:**
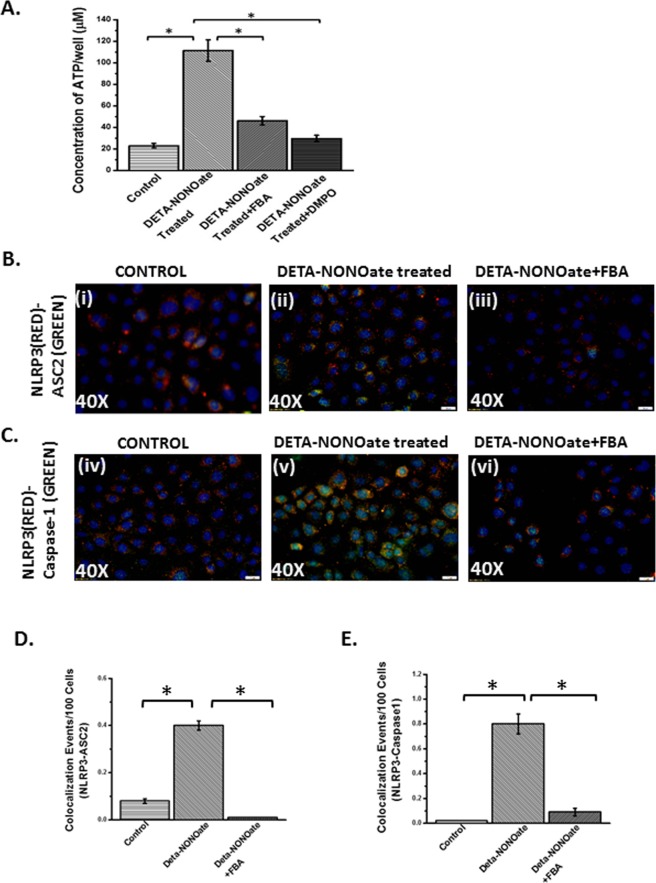
(**A**) ATP assay was performed using the Intestinal Epithelial cells (IEC-6) cell supernatants for the groups Control, DETA-NONOate treated, DETA-NONOate treated+ FBA and DETA-NONOate treated + DMPO, to study the concentration of ATP per well. (n = 3, analysis from three samples) (*p < 0.05). Intestinal epithelial (IEC-6) cell line was used as control (CONTROL), one group was treated with the supernatant of IEC-6 cell line that was treated with NO-donor (DETA-NONOate), another group of cells were blocked by FBA and then was treated with the NO-donor (DETA-NONOate) (DETA-NONOate + FBA). The cell groups were used for immunofluorescence imaging. (**B**) (i–iii) Immunofluorescence images depicting NLRP3 (red)-ASC2 (green) colocalization and (**C**) (iv–vi) NLRP3 (red)- Caspase-1 (green) colocalization, counterstained with DAPI (blue) in CONTROL, DETA-NONOate,DETA-NONOate + FBA, group of IEC-6 cells. Images were taken at 20X magnification. (**D**) Morphometric analysis of NLRP3-ASC2 and (**E**) NLRP3-Caspase1 colocalized events. Y-axis shows % positive immunoreactive area (% ROI) (n = 5, analysis from five separate microscopic fields) (*p < 0.05).

Peroxynitrite generation requires a diffusion-controlled reaction of nitric oxide and superoxide released from NOX-2 or a subsequent enzyme capable of releasing the same^36^. We also showed increased nitric oxide release in cells that were incubated with leptin + microcystin. The NO release was significantly higher in the leptin + Microcystin (Leptin+MC) group when compared with either cells only group or cells incubated with leptin alone (P < 0.05) (Fig. 14B). To show that nitric oxide is crucial for the mechanisms of peroxynitrite generation and NLRP3 inflammasome activation we used a NO donor DETANONOATE^37^. Since extracellular ATP is one of the prime ligands for inflammasome activation, we studied the release of ATP from the intestinal epithelial cells following microcystin exposure in the presence of NO donor, NOX-2 inhibitor apocynin and peroxynitrite scavenger FBA. Results showed that extracellular ATP release was significantly increased in the presence of the NO donor and was consistent with our previous data that show leptin primed cells incubated with microcystin released NO (P < 0.05)(Fig. 15A). FBA or oxyradical scavenger and nitrone spin trap DMPO significantly decreased ATP levels, when compared to NO donor treatment group (P < 0.05) (Fig. 15A).

DETANONOATE, the NO donor significantly increased inflammasome activation (NLRP3-ASCII colocalization, Fig. 15Bi–iii,D) and recruitment of Caspase-1 in the inflammasome complex (Fig. 15Civ–vi,E)(p < 0.05) while the administration of FBA, a peroxynitrite scavenger significantly decreased the NLRP3 activation and Caspase-1 recruitment suggesting NOX2-derived superoxide and nitric oxide in the cellular microenvironment caused peroxynitrite generation and NLRP3 activation.

## Discussion

We report a novel mechanism of environmental low dose microcystin exposure-induced exacerbation of intestinal inflammatory pathology in an underlying NAFLD. Microcystin exposure independently altered and worsened gut dysbiosis at the phylum, order, family and genus levels. Though the altered dysbiosis bore the signature of worsened NAFLD, new information related to significant increases in the order Clostridiales, family clostridiaceae and genus blautia were observed. Interestingly, microcystin alone only increased the abundance of these bacteria marginally suggesting that an underlying asymptomatic NAFLD helped exacerbate the effect of microcystin on the digestive tract. It is important to note that increases in the abundance of Clostridiales, Clostridiaceae, Lachnospiraceae and Intestinibacter has been indicative of severe intestinal disturbances in humans^38,39^. Increased abundance of Clostridiaceae has been associated with increased incidences of IBD-RA (Rheumatoid Arthritis)^40^. Our finding of increased abundance of Clostridiales, Lachnospiraceae and the genus Intestinibacter are recognized as strong biomarkers of early onset of Chron’s disease, an intestinal inflammatory condition with no discrete therapeutic regimen^41^. The result reported in our study where mice with asymptomatic NAFLD were exposed to microcystin provides a strong basis that environmental exposure in patients with NAFLD carry an immense risk of progressing to a more severe phenotype of the disease. Importantly as mentioned before, NAFLD is now considered as a pandemic and is rapidly being reported not only in the developed world but encompasses more populations in the developing countries of India and China^42^. As more and more water bodies across the world report existence of harmful algal blooms and increased cyanobacterial abundance, it is not difficult to predict that our findings lay a strong basis for defining the risk factors in susceptible populations worldwide especially intestinal comorbidities of an existing disease^43^. These exacerbations can be difficult to treat and will affect health care costs tremendously. Our results, though show a strong association of microbial changes with microcystin exposure does not rule out its role as a hepatotoxic agent^44^. However, our data strongly suggest that microcystin exposure in low doses can influence the microbiome in parallel to its toxic effects on the liver.

Our results show that a change in gut microbiome following microcystin exposure was associated strongly with intestinal epithelium erosion, crypt abscess and granulated cells, hallmarks of intestinal inflammatory disease. The results assume significance since NAFLD severity is often associated with inflammatory bowel disease, where the cause and pathology remained unclear^7,10^. On the contrary, many reports show that IBD and NAFLD severity coexist and bear the same pathological signatures that we show in our model study suggesting that environmental microcystin exposure can be a possible cause to trigger disease severity and coexistence. Mechanistically we also report the activation of NLRP3-inflammasome and loss of gut barrier integrity, a prime pathological event in many inflammatory disease conditions that involve the intestine^26^. Gut barrier integrity is also crucial for prevention of leaching of intestinal damage associated molecular patterns capable of eliciting low lying inflammation in various organ systems including the liver^45^. Our results of increased claudin-2 and parallel decreases in Occludin followed by increased endotoxin in the circulation indicate a strong possibility of gut leaching following microcystin exposure and microbial dysbiosis. The results also provide a strong rationale for the role of NLRP3 inflammasome as a mechanism for the downstream inflammatory events following microcystin-associated dysbiosis in NAFLD.

Several reports tie microbial dysbiosis to inflammatory events in the gut but the pathways connecting these two significant events remain obscure. We have shown previously that inflammasome activation or the pathways leading to proinflammatory disease conditions in the intestine with or without NAFLD might implicate the role of redox signaling where NADPH oxidase (NOX-2) plays an important role^29,45^. Strikingly, the dysbiosis that preceded inflammasome formation was also associated strongly with a significant increase inNOX-2 activation and tyrosine radical formation in our model. We found that the lactate producing bacteria in the gut (Lactobacillus and Enterococcus) showed a >2 fold increase in Microcystin exposure. Strikingly, lactate produced in drosophila is known to activate NOX-2^46^. The study by Latsenko *et al*. showed that a null mutation in Drosophila PGRP-SD was associated with overgrowth of Lactobacillus plantarum in the fly gut and a shortened lifespan and L. plantarum-derived lactic acid triggered the activation of the intestinal NADPH oxidase^46^. Though no such study has been reported in disease models, we justifiably assume that increases in lactate producing bacteria (Lactobacillus and Enterococcus) following microcystin exposure might trigger the increased lactate needed to activate NOX-2. The result is very likely correlative at this point but a measurement of intestinal lactatemight put an end to this speculation and remains a minor limitation in the present study.

The involvement of NOX-2-derived reactive oxygen species in inflammatory phenotype was also proven by the use of NOX-2-knockout mice that had a deletion of the p47 phox gene. NOX-2 knockout mice showed decreased inflammation, NLRP3 activation and decreased gut leaching, thus confirming its role in microbial derived intestinal tissue injury. The result also confirmed that NOX-2 interfered with inflammation downstream of microbial dysbiosis since p47 phox knockout mice did not show the pattern of microbial changes akin to what is reported following microcystin exposure.

We have shown previously that NOX-2 mediates the redox signaling process in inflammation via the generation of peroxynitrite^30^. Notably, peroxynitrite species are formed following a diffusion-controlled reaction with superoxide (likely derived from NOX-2 and nitric oxide^11^. We identified that NOX-2 activation following microcystin exposure and gut dysbiosis led to peroxynitite generation, an observation confirmed further with the use of specific peroxynitite scavenger phenyl boronic acid (FBA)^47^.

Possible bias and limitations of the study. There is scant evidence of microcystin exposure from cyanobacterial blooms and how it affects disease development. Our animal model mimics a hypothetical scenario where environmental microcystin exposure to populations having existing NAFLD. Though clinically, it is an arduous task to differentiate the environmental effect in the progression of NAFLD, our study attempts to explain the pathophysiology of such a scenario where increased harmful algal blooms or exposure to microcystin can increase the risk of NAFLD progression to NASH. Microcystin exposure in our study was based on a cumulative sub-chronic model with only two weeks. This particular dose, used in our study was far from adequate in predicting the different types and nature of exposures that humans might receive. A recent study Greer B *et al*. describe a porcine model of microcystin exposure which might be a good model to pursue for detection of pathology in organ systems{Greer, 2018 #2632}^48^. Future studies with diverse exposure regiment needs to be done to assess the severity of disease progression. Studies in rodent models of NASH have been paramount to the elucidation of the pathophysiology and drug discovery to understand and treat humanNASH knowing fully well that the disease is presented late and there are no clinical biomarkers of this disease. Thus, it is justifiable to assume that our present study will help in advancing our knowledge in understanding the environmental effects inNASH progression. Further, it is known that mice and humans share significant similarities in anatomy and physiology. A recent study also compares the details of the mice and human microbiota {Nguyen, 2015 #2631}^49^. The study also finds that there are atleast 79 genera occurring in both human and mouse gut microbiotas. The relative abundances of most of the dominant genera in mouse and human are quite different. Also, Clostridium, Bacteroides and Blautia, on the contrary, have a similar relative abundance in both organisms though lactobacillus seemed to be very abundant only in the murine species. Despite this, the major gut microbiota shifts that have been observed in different obesity, NAFLD and colitis mouse models are similar to those found in human IBD studies.

Thus, in summary, our studies show a novel pathway of microcystin-induced exacerbation of NAFLD comorbidities via gut dysbiosis and redox signaling. The study also supports an emerging hypothesis that chronic microcystin exposure as might happen through drinking water contaminated with cyanobacterial toxins might aid in disease complexity and pathogenesis. The present study also can be used as a model for microcystin risk assessment in cohorts who are already plagued with asymptomatic and silent disease conditions like NAFLD and/or chronic conditions like obesity, Type 2 Diabetes and hypertension. Though the present study is conducted in a preclinical set up, future studies need to be conducted in human cohorts that associate exposure of cyanobacterial toxins and disease conditions with parallel insights to changes in gut microbiome.

## Data Availability

All data generated or analyzed during this study are included in this published article. Any additional datasets generated during and/or analyzed during the current study are available from the corresponding author on reasonable request.

## References

[1] Augustin S, Graupera I, Caballeria J (2017). & ennombre del grupo de trabajo sobre de la Societat Catalana de, D. Non-alcoholic fatty liver disease: A poorly known pandemic. Med Clin (Barc).

[2] El-Zayadi AR (2008). Hepatic steatosis: a benign disease or a silent killer. World journal of gastroenterology: WJG.

[3] Puoti C, Elmo MG, Ceccarelli D, Ditrinco M (2017). Liver steatosis: The new epidemic of the Third Millennium. Benign liver state or silent killer? Eur J Intern Med.

[4] Bohinc BN, Diehl AM (2012). Mechanisms of disease progression inNASH: new paradigms. Clinics in liver disease.

[5] Tilg H, Moschen AR (2014). Evolving therapies for non-alcoholic steatohepatitis. Expert opinion on drug discovery.

[6] Younossi Zobair M. (2019). Non-alcoholic fatty liver disease – A global public health perspective. Journal of Hepatology.

[7] Wieser V, Gerner R, Moschen AR, Tilg H (2013). Liver complications in inflammatory bowel diseases. Digestive diseases (Basel, Switzerland).

[8] Mahamid M, Yassin T, Abu Elheja O, Nseir W (2017). Association between Fatty Liver Disease and Hyperplastic Colonic Polyp. Isr Med Assoc.

[9] Ahmed M (2015). Non-alcoholic fatty liver disease in 2015. World J Hepatol.

[10] Reddy SK, Zhan M, Alexander HR, El-Kamary SS (2013). Nonalcoholic fatty liver disease is associated with benign gastrointestinal disorders. World journal of gastroenterology: WJG.

[11] Chao CY (2016). Co-existence of non-alcoholic fatty liver disease and inflammatory bowel disease: A review article. World journal of gastroenterology: WJG.

[12] Wahlang B (2013). Toxicant-associated steatohepatitis. Toxicologic pathology.

[13] Seth Ratanesh Kumar, Kumar Ashutosh, Das Suvarthi, Kadiiska Maria B., Michelotti Gregory, Diehl Anna Mae, Chatterjee Saurabh (2013). Environmental Toxin–Linked Nonalcoholic Steatohepatitis and Hepatic Metabolic Reprogramming in Obese Mice. Toxicological Sciences.

[14] Arciello M (2013). Environmental pollution: a tangible risk for NAFLD pathogenesis. International journal of molecular sciences.

[15] Trevino LS, Katz TA (2018). Endocrine Disruptors and Developmental Origins of Nonalcoholic Fatty Liver Disease. Endocrinology.

[16] Schmidt JR, Wilhelm SW, Boyer GL (2014). The fate of microcystins in the environment and challenges for monitoring. Toxins (Basel).

[17] Gehringer MM, Wannicke N (2014). Climate change and regulation of hepatotoxin production in Cyanobacteria. FEMS microbiology ecology.

[18] Diez-Quijada L (2019). Microcystin-RR: Occurrence, content in water and food and toxicological studies. A review. Environ Res.

[19] He J (2017). Prolonged exposure to low-dose microcystin induces nonalcoholic steatohepatitis in mice: a systems toxicology study. Arch Toxicol.

[20] Weber N (2018). Nephele: a cloud platform for simplified, standardized and reproducible microbiome data analysis. Bioinformatics.

[21] Sharpton Suzanne R., Ajmera Veeral, Loomba Rohit (2019). Emerging Role of the Gut Microbiome in Nonalcoholic Fatty Liver Disease: From Composition to Function. Clinical Gastroenterology and Hepatology.

[22] Bernstein CN, Forbes JD (2017). Gut Microbiome in Inflammatory Bowel Disease and Other Chronic Immune-Mediated Inflammatory. Diseases. Inflamm Intest Dis.

[23] Ramsey, M., Hartke, A. & Huycke, M. in *Enterococci: From Commensals to Leading Causes of Drug Resistant Infection* (eds Gilmore, M. S., Clewell, D. B., Ike, Y. & Shankar, N.) (2014). 24649510

[24] Shen F (2017). Gut microbiota dysbiosis in patients with non-alcoholic fatty liver disease. Hepatobiliary & pancreatic diseases international: HBPD INT.

[25] Ozaki R (2018). Histological Risk Factors to Predict Clinical Relapse in Ulcerative Colitis With Endoscopically Normal Mucosa. J Crohns Colitis.

[26] Mao L, Kitani A, Strober W, Fuss IJ (2018). The Role of NLRP3 and IL-1beta in the Pathogenesis of Inflammatory Bowel Disease. Frontiers in immunology.

[27] Liu Q, Zhang D, Hu D, Zhou X, Zhou Y (2018). The role of mitochondria inNLRP3 inflammasome activation. Mol Immunol.

[28] Pang J (2017). Significant positive association of endotoxemia with histological severity in 237 patients with non-alcoholic fatty liver disease. Alimentary pharmacology & therapeutics.

[29] Alhasson F (2017). Altered gut microbiome in a mouse model of Gulf War Illness causes neuroinflammation and intestinal injury via leaky gut and TLR4 activation. PloS one.

[30] Das Suvarthi, Alhasson Firas, Dattaroy Diptadip, Pourhoseini Sahar, Seth Ratanesh Kumar, Nagarkatti Mitzi, Nagarkatti Prakash S., Michelotti Gregory A., Diehl Anna Mae, Kalyanaraman Balaraman, Chatterjee Saurabh (2015). NADPH Oxidase–Derived Peroxynitrite Drives Inflammation in Mice and Human Nonalcoholic Steatohepatitis via TLR4-Lipid Raft Recruitment. The American Journal of Pathology.

[31] Das S (2013). Purinergic receptor X7 is a key modulator of metabolic oxidative stress-mediated autophagy and inflammation in experimental nonalcoholic steatohepatitis. American journal of physiology. Gastrointestinal and liver physiology.

[32] Chatterjee S (2013). Leptin is key to peroxynitrite-mediated oxidative stress and Kupffer cell activation in experimental non-alcoholic steatohepatitis. J Hepatol.

[33] Mangan MSJ (2018). Targeting the NLRP3 inflammasome in inflammatory diseases. *Nature reviews*. Drug discovery.

[34] Cernea S, Roiban AL, Both E, Hutanu A (2018). Serum leptin and leptin resistance correlations with NAFLD in patients with type 2 diabetes. Diabetes Metab Res Rev.

[35] Roh YS, Seki E (2018). Chemokines and Chemokine Receptors in the Development of NAFLD. Advances in experimental medicine and biology.

[36] Das S (2015). NADPH Oxidase-Derived Peroxynitrite Drives Inflammation in Mice and HumanNonalcoholic Steatohepatitis via TLR4-Lipid Raft Recruitment. The American journal of pathology.

[37] Seth RK (2015). M1 polarization bias and subsequent nonalcoholic steatohepatitis progression is attenuated by nitric oxide donor DETA NONOate via inhibition of CYP2E1-induced oxidative stress in obese mice. The Journal of pharmacology and experimental therapeutics.

[38] Gevers D (2014). The treatment-naive microbiome innew-onset Crohn’s disease. Cell Host Microbe.

[39] Wingfield, B., Coleman, S., McGinnity, T. M. & Bjourson, A. Robust Microbial Markers for Non-Invasive Inflammatory Bowel Disease Identification. *IEEE/ACM Trans Comput Biol Bioinform*, 10.1109/TCBB.2018.2831212 (2018). 10.1109/TCBB.2018.2831212 29994028

[40] Muñiz Pedrogo David A, Chen Jun, Hillmann Benjamin, Jeraldo Patricio, Al-Ghalith Gabriel, Taneja Veena, Davis John M, Knights Dan, Nelson Heidi, Faubion William A, Raffals Laura, Kashyap Purna C (2018). An Increased Abundance of Clostridiaceae Characterizes Arthritis in Inflammatory Bowel Disease and Rheumatoid Arthritis: A Cross-sectional Study. Inflammatory Bowel Diseases.

[41] Tomasello G (2011). From gut microflora imbalance to mycobacteria infection: is there a relationship with chronic intestinal inflammatory diseases?. Ann Ital Chir.

[42] Younossi Zobair, Tacke Frank, Arrese Marco, Chander Sharma Barjesh, Mostafa Ibrahim, Bugianesi Elisabetta, Wai-Sun Wong Vincent, Yilmaz Yusuf, George Jacob, Fan Jiangao, Vos Miriam B. (2019). Global Perspectives on Nonalcoholic Fatty Liver Disease and Nonalcoholic Steatohepatitis. Hepatology.

[43] Paerl HW (2016). Mitigating cyanobacterial harmful algal blooms in aquatic ecosystems impacted by climate change and anthropogenic nutrients. Harmful Algae.

[44] Dawson RM (1998). The toxicology of microcystins. Toxicon.

[45] Chandrashekaran V (2017). HMGB1-RAGE pathway drives peroxynitrite signaling-induced IBD-like inflammation in murine nonalcoholic fatty liver disease. Redox biology.

[46] Iatsenko I, Boquete JP, Lemaitre B (2018). Microbiota-Derived Lactate Activates Production of Reactive Oxygen Species by the Intestinal NADPH Oxidase Nox and Shortens Drosophila Lifespan. Immunity.

[47] Zielonka J, Sikora A, Joseph J, Kalyanaraman B (2010). Peroxynitrite is the major species formed from different flux ratios of co-generated nitric oxide and superoxide: direct reaction with boronate-based fluorescent probe. J Biol Chem.

[48] Greer B, Meneely JP, Elliott CT (2018). Uptake and accumulation of Microcystin-LR based on exposure through drinking water: An animal model assessing the human health risk. Scientific reports.

[49] Nguyen TL, Vieira-Silva S, Liston A, Raes J (2015). How informative is the mouse for human gut microbiota research?. Dis Model Mech.

